# Towards Improved Humidity Sensing Nanomaterials via Combined Electron and NH_3_ Treatment of Carbon-Rich FEBID Deposits

**DOI:** 10.3390/nano12244455

**Published:** 2022-12-15

**Authors:** Hannah Boeckers, Petra Swiderek, Markus Rohdenburg

**Affiliations:** 1 Institute for Applied and Physical Chemistry, University of Bremen, Leobener Str. 5, 28359 Bremen, Germany; 2 Wilhelm-Ostwald-Institute for Physical and Theoretical Chemistry, Leipzig University, Linnéstr. 2, 04103 Leipzig, Germany

**Keywords:** focused electron beam induced deposition, carbonaceous nanomaterials, humidity sensing, nitrogen incorporation, ammonia

## Abstract

Focused Electron Beam Induced Deposition (FEBID) is a unique tool to produce nanoscale materials. The resulting deposits can be used, for instance, as humidity or strain sensors. The humidity sensing concept relies on the fact that FEBID using organometallic precursors often yields deposits which consist of metal nanoparticles embedded in a carbonaceous matrix. The electrical conductivity of such materials is altered in the presence of polar molecules such as water. Herein, we provide evidence that the interaction with water can be enhanced by incorporating nitrogen in the deposit through post-deposition electron irradiation in presence of ammonia (NH_3_). This opens the perspective to improve and tune the properties of humidity sensors fabricated by FEBID. As a proof-of-concept experiment, we have prepared carbonaceous deposits by electron irradiation of adsorbed layers of three different precursors, namely, the aliphatic hydrocarbon *n*-pentane, a simple alkene (2-methyl-2-butene), and the potential Ru FEBID precursor bis(ethylcyclopentadienyl)ruthenium(II). In a subsequent processing step, we incorporated C-N bonds in the deposit by electron irradiation of adsorbed NH_3_. To test the resulting material with respect to its potential humidity sensing capabilities, we condensed sub-monolayer quantities of water (H_2_O) on the deposit and evaluated their thermal desorption behavior. The results confirm that the desorption temperature of H_2_O decisively depends on the degree of N incorporation into the carbonaceous residue which, in turn, depends on the chemical nature of the precursor used for deposition of the carbonaceous layer. We thus anticipate that the sensitivity of a FEBID-based humidity sensor can be tuned by a precisely timed post-deposition electron and NH_3_ processing step.

## 1. Introduction

Focused Electron Beam Induced Deposition (FEBID) is a versatile nanofabrication tool that can be employed to produce arbitrarily shaped structures on surfaces by decomposition of organometallic precursors under a tightly focused high-energy electron beam [1,2]. Typically, the purity of FEBID deposits is low due to incomplete precursor fragmentation under electron impact. Material from the organic ligands becomes embedded in the deposit leading to high carbon contents. Transmission electron microscopy (TEM) has revealed that FEBID deposits often consist of metal grains incorporated in a carbonaceous matrix. This has been extensively studied for FEBID deposits produced from trimethyl(methylcyclopentadienyl)platinum(IV) (MeCpPtMe_3_) for which a nanogranular structure with a Pt content of 10–15% [3] and a typical grain diameter of 2–3 nm [4] has been reported. Although this impedes applications requiring the properties of a purely metallic material such as high electrical conductivity, carbonaceous FEBID deposits have interesting prospects in, e.g., humidity [4] or strain sensing [5]. Both sensing concepts rely on the fact that the electrical resistance of the material depends on the probability of electron tunneling and thus on the distance between the metal grains [4]. While strain can mechanically reduce or increase the grain distance thus enhancing or impeding the tunnel current [5], water (H_2_O) molecules from the background gas can adsorb on the deposit causing an increase in the dielectric constant within the material [4]. This increases tunneling probabilities and thus also the tunnel current that can be measured when a voltage is applied across the deposit. Note that adsorption of nonpolar molecules such as CO_2_ or O_2_ from the background gas has virtually no impact on the dielectric constant of the deposit so that the electron tunneling probability is not affected [4]. Such sensors based on carbonaceous deposits are thus specifically suited for selective humidity sensing. Although FEBID-based humidity sensors are not as widely implemented as other devices based, e.g., on optical scattering in microarray polymer materials [6], such nanoscale sensors could possibly find wider application when their sensitivity is increased.

The extent of H_2_O adsorption on a given surface depends on the strength of the interactions between H_2_O and the surface. Thermal desorption spectrometry (TDS) can probe these interactions in terms of desorption temperatures. In the present study, we employ TDS to investigate the binding strength of H_2_O to carbonaceous materials and, in particular, to demonstrate how this binding can be modified. The experiments were performed at sub-monolayer coverages of H_2_O to avoid the formation of multilayer adsorbates for which the desorption temperature is rather governed by interactions between the individual H_2_O molecules. The adsorption of H_2_O on pure hydrocarbon films based on Van der Waals interactions has been reported to be rather weak. For instance, it was observed that sub-monolayer H_2_O films desorb at a temperature of 139 K from rough carbon surfaces [7], i.e., approximately 15 K below the multilayer desorption temperature. A similarly low desorption temperature was observed for sub-monolayer H_2_O films adsorbed on graphite [8,9]. This is an unusual behavior because the desorption of sub-monolayers is typically observed at higher temperatures than the multilayer due to the stronger interactions between the adsorbate and the surface as compared to those between the molecules within the multilayer. This particularly low desorption temperature of H_2_O from a purely carbonaceous surface indicates that H_2_O binding on carbon-rich FEBID deposits may as well be rather weak. This rationalizes why the sensitivity of conventional sensors based on FEBID from MeCpPtMe_3_ as precursor is limited. For instance, it was reported that an increase in the humidity level from 5% to 78% causes an increase in the relative tunnel current passing through the nanosensor by a mere 10% [4]. Having the above-described humidity sensing concept in mind, a stronger adsorption of the analyte H_2_O on the sensor surface is beneficial leading, in the ideal case, to a situation where even lower amounts of water would produce a sufficient surface coverage to cause a measurable sensor signal.

**Figure 1:**
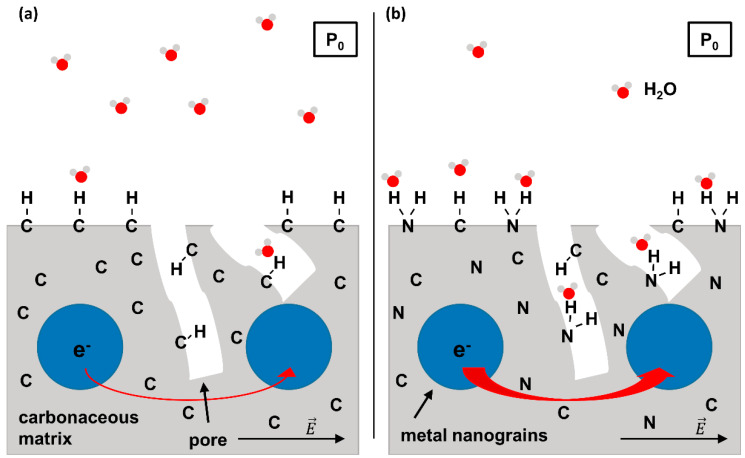
Scheme of the working principle of a FEBID-based humidity sensor. (**a**) Conventional concept relying on metal-carbon nanocomposite material (i.e., metal nanograins embedded into a porous carbonaceous matrix). When applying an external voltage to the sensor, electrons can tunnel between the metal grains due to the electric field $\vec{E}$. The tunnel probability is enhanced by polar molecules that adsorb on the surface of the sensor. However, adsorption of polar H_2_O molecules on a purely carbonaceous surface due to van der Waals interactions is weak. (**b**) FEBID-based humidity sensor with enhanced sensitivity due to N incorporation. The possibility of H bond formation between H_2_O and surface N species allows for a stronger adsorption so that at a given H_2_O partial pressure P_0_, more H_2_O molecules will adsorb as compared to the sensor shown in (**a**). This results in an increased tunnel current as symbolized by the thicker red arrow visualizing electron transfer between metal grains. Note that the partial pressure of H_2_O (P_0_) is supposed to be identical in (**a**,**b**).

**Figure 2:**
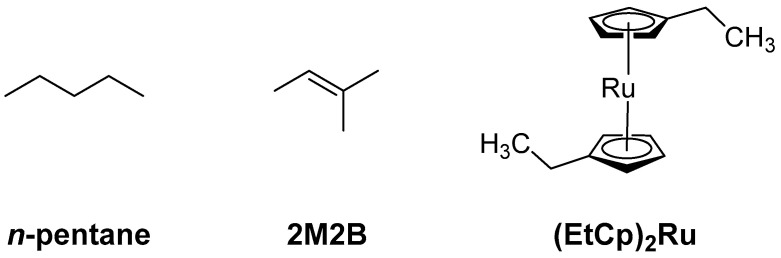
Precursor molecules used in the present study to prepare adsorbate layers that were converted to carbonaceous deposits by electron irradiation for subsequent N incorporation by electron irradiation in presence of NH_3_.

A strategy to improve the H_2_O binding capabilities of carbonaceous FEBID deposits is to incorporate stronger adsorption sites within the existing carbonaceous matrix. As a first step towards this goal we demonstrated recently that a material with the composition of a carbon nitride (C_3_N_4_) with embedded Ru grains is obtained when deposits fabricated by FEBID from bis(ethylcylcopentadienyl)ruthenium(II) ((EtCp)_2_Ru) are subject to post-deposition treatment by electron irradiation in presence of ammonia (NH_3_) [10]. Such incorporation of nitrogen into the carbonaceous matrix of the deposit is anticipated to provide stronger binding sites for H_2_O such as polar -NH_2_ groups and thus an enhanced sensing performance as depicted in Figure 1. In fact, amorphous C_3_N_4_ was already suggested as a cheap and easily accessible gas sensor [11]. Motivated by these results, we have investigated the incorporation of N into deposits produced by electron irradiation from three hydrocarbon-containing compounds, namely aliphatic *n*-pentane, the unsaturated compound 2-methyl-2-butene (2M2B), and the previously studied FEBID precursor (EtCp)_2_Ru (Figure 2). Using these deposits, the effect of N uptake on the binding of H_2_O was revealed.

While metal-free hydrocarbons cannot be used for the fabrication of actual sensing materials based on tunneling between metal grains, the increasing complexity of the different compounds from a fully saturated hydrocarbon to a metal-containing precursor reveals if the incorporation of N depends on the chemical structure of a precursor and how the latter affects the capability of the modified deposit to bind H_2_O. To this end, we have employed, along with TDS, Auger electron spectroscopy (AES) in the clean environment of an ultrahigh vacuum (UHV) chamber to monitor the changes in the elemental composition and, in particular, the incorporation of N into the deposits during electron beam processing in presence of NH_3_. The study is designed to confirm that the H_2_O binding capability of deposits prepared by FEBID can be tuned by incorporating N into the carbonaceous matrices of the deposits. The results also provide guidance towards the selection of suitable precursors for the fabrication of FEBID-based humidity sensors and thus serve as a next step towards the direct-write fabrication of nanoscale humidity sensors with tunable sensitivity.

## 2. Materials and Methods

### 2.1. Chemicals

*n*-Pentane (Riedel-de Haën, Seelze, Germany, purity 99%), 2-methyl-2-butene (2M2B, Sigma Aldrich, St. Louis, MO, USA, purity 99%), and bis(ethylcylcopentadienyl)ruthenium(II) ((EtCp)_2_Ru, STREM Chemicals, Newburyport, MA, USA, purity 98%, 99.9% Ru) were used without further purification as precursors for the preparation of carbonaceous deposits. All precursors were degassed by repeated freeze-pump-thaw cycles and kept under vacuum and in the dark throughout all experiments. Ammonia (NH_3_, Linde GmbH, Pullach, Germany, purity > 99%) was used for incorporation of N in the deposits and used as received.

### 2.2. UHV Setup

Deposit preparation, post-deposition NH_3_, and electron beam treatment, as well as H_2_O desorption experiments were performed in an UHV chamber operated at a base pressure of about 10^−10^ mbar. Deposition was carried out on a polycrystalline Ta substrate held at 110 K by liquid nitrogen cooling. The sample temperature is controlled by resistive heating of two thin Ta ribbons spot-welded to the thicker Ta sheet and is measured using a type E thermocouple press-fitted to the Ta substrate. The setup is equipped with a quadrupole mass spectrometer (QMS) residual gas analyzer (300 amu, Stanford Research Systems, Sunnyvale, CA, USA) with electron impact ionization at 70 eV, a commercial flood gun (SPECS FG 15/40, SPECS Surface Nano Analysis GmbH, Berlin, Germany) for electron irradiation in the range of E_0_ = 1–500 eV, an Auger electron spectrometer (STAIB DESA 100, STAIB INSTRUMENTS GmbH, Langenbach, Germany), and a sputter gun for surface cleaning.

### 2.3. Deposit Preparation, Thermal Desorption Spectrometry (TDS), and Electron-Stimulated Desorption (ESD)

Deposits were prepared in line with previously reported procedures [12,13]. Prior to each experiment, the Ta substrate was sputter-cleaned using Ar^+^ ions at a kinetic energy of 3 keV until the Ta signals were clearly visible in AES and any other signals, in particular, remaining O and C signals had disappeared. Immediately before each deposition, adsorbed volatile compounds from the residual gas were further removed by annealing to 450 K. The precursors were then condensed on the Ta sheet at temperatures between 100 K and 110 K over a period of a few minutes. This was performed by introducing the precursor vapor via a gas handling manifold consisting of precision leak valves and a small, calibrated volume where the absolute pressure is measured with a capacitance manometer. For each film deposition, a calibrated amount of vapor stated herein as pressure drop in the manifold was leaked via a stainless-steel tube opening onto the Ta substrate.

TDS was performed to measure the desorption temperature of H_2_O and to estimate the thickness of precursor layers that were adsorbed at 110 K for subsequent electron irradiation as described in detail in Section 3.1. In TDS experiments, the QMS was used to monitor characteristic *m*/*z* ratios of desorbing species during application of a temperature ramp of 1 K/s to the sample. TDS data acquired for varying amounts of vapor provide information on the evolution from sub-monolayer coverage to a multilayer adsorbate as illustrated most clearly for the case of *n*-pentane (Supporting Information, Figure S1). While low vapor doses yield a monolayer signal in the temperature range between 150 K and 200 K, higher doses produce a continuously increasing multilayer desorption peak with well-defined maximum at 115 K. The multilayer peak starts to emerge between vapor doses of 0.22 mTorr and 0.35 mTorr. These values thus provide an estimate of the amount of vapor that is required to form a monolayer of *n*-pentane on the supporting Ta substrate as 0.29 mTorr ± 25%. Due to a somewhat higher substrate temperature, the transition from the monolayer to the multilayer was less clear in the case of 2M2B (Supporting Information, Figure S2a). Therefore, the estimate based on the appearance of the multilayer desorption signal that points to monolayer saturation at vapor doses between 0.25 mTorr and 0.6 mTorr was further supported by data for larger amounts of vapor for which the peak height of the multilayer peak was plotted as function of vapor dose (Supporting Information, Figure S2b). The extrapolation of the peak height to a baseline intensity level together with that visual inspection of the data for lower vapor doses (Supporting Information, Figure S2a) then yields that a vapor dose of 0.43 mTorr ± 40% is required for monolayer formation. The vapor dose required to produce a monolayer of (EtCp)_2_Ru was estimated previously to correspond to a pressure drop in the range of 0.20−0.30 mTorr [12,13], which translates to a value of 0.25 mTorr ± 20%. The thickness of the adsorbed layers is given in numbers of monolayers (ML) as estimated from the vapor dose and these monolayer estimates. The margin of error in the preparation of adsorbed precursor layers is the same for all experiments and therefore not explicitly stated in the following. Note that the actual thickness of a monolayer of *n*-pentane and 2M2B can be estimated from the densities of the compounds [14,15] as 5.7 Å and 5.6 Å, respectively. The thickness of a monolayer of (EtCp)_2_Ru is taken to be similar to that of MeCpPtMe_3_ which has been reported as 0.93 nm [16].

The adsorbed precursor layers were converted to crosslinked carbonaceous deposits by electron irradiation as described in detail in Section 3.1. This procedure typically took approximately 60 min. The QMS was used during this step to monitor electron-stimulated desorption (ESD) of neutral species from the surface.

For each step of the N incorporation procedure, an amount of NH_3_ vapor corresponding to a pressure drop of 4 mTorr in the manifold was leaked onto the deposits in line with a protocol established earlier [10]. The resulting surface coverage of NH_3_ was estimated previously to be at least within the monolayer regime [12,13]. Finally, we refer to the discussion in Section 3.2 regarding the amount of H_2_O vapor required to form an adsorbate within the monolayer regime.

### 2.4. Auger Electron Spectroscopy (AES)

All AE spectra were recorded with a STAIB DESA 100 Auger electron spectrometer (STAIB INSTRUMENTS GmbH, Langenbach, Germany) using an incident electron energy of 5 keV in pulse counting acquisition mode. Derivative AE spectra were obtained by numerically differentiating the collected spectra and applying a Savitzky–Golay filter for smoothing.

### 2.5. Reflection Absorption Infrared Spectroscopy (RAIRS)

The infrared beam of a commercial RAIR spectrometer (IFS 66v/S, Bruker Optics GmbH, Ettlingen, Germany) is guided through a KBr window into the UHV chamber, allowing for vibrational spectroscopy of adsorbates on the Ta sheet under grazing incidence conditions. The RAIR spectrometer is equipped with a liquid nitrogen-cooled MCT detector with a sensitivity limit down to 750 cm^−1^. RAIR spectra were collected from 4000 to 750 cm^−1^ with a resolution of 4 cm^−1^ by averaging 400 single scans. The optics system was continuously purged with N_2_ to eliminate contributions of residual vapors to the RAIR spectra.

## 3. Results

### 3.1. General Procedures

**Figure 3:**
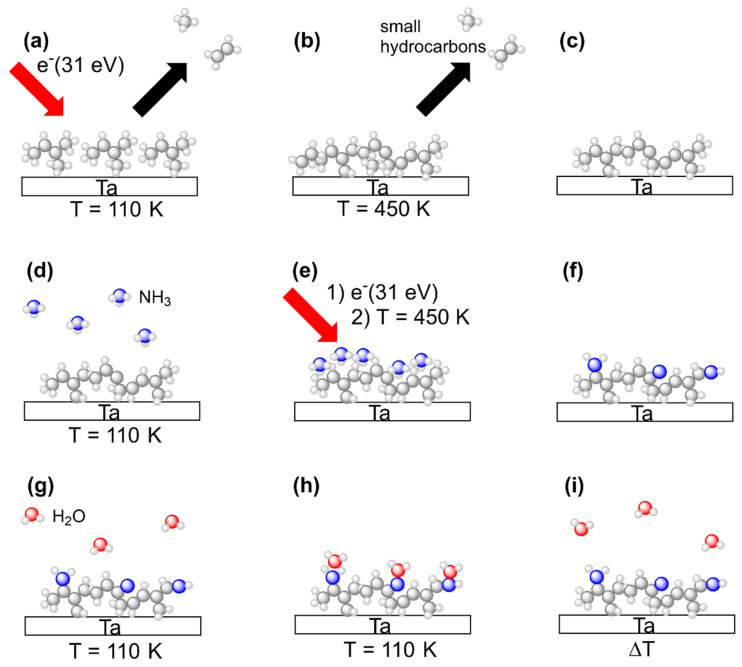
Overview of experiments to deposit (top row) and process (middle row) carbonaceous surface layers as well as to characterize their H_2_O binding capabilities (bottom row). The scheme shows all process steps for the example of 2M2B but the same experimental procedures also apply to (EtCp)_2_Ru and *n*-pentane. (**a**) Multilayer films of the precursor condensed onto a cooled Ta sheet held at T = 110 K are exposed to electrons with an energy of E_0_ = 31 eV. Volatile irradiation products are simultaneously monitored by the QMS in an ESD experiment. (**b**) Subsequent annealing of the Ta sheet to T = 450 K is employed to remove less volatile irradiation products and unreacted precursor molecules from the crosslinked surface layer. (**c**) As a result, a crosslinked carbonaceous residue is obtained on the Ta surface. (**d**) NH_3_ is condensed in multilayer coverages onto the carbonaceous surface layer cooled again to T = 110 K. (**e**) Electron irradiation at E_0_ = 31 eV followed by annealing of the sample to T = 450 K is employed to induce N incorporation reactions and subsequently remove unreacted NH_3_ as well as volatile reaction products of NH_3_ with the surface layer. (**f**) The crosslinked residue now contains N as confirmed by AES. (**g**) H_2_O is condensed in sub-monolayer coverages onto the NH_3_-processed carbonaceous surface layer held at T = 110 K. (**h**) H_2_O can form hydrogen bonds to the N-containing groups in the layer so that an increase in adsorption energy is caused. (**i**) In a final TDS experiment, H_2_O is thermally desorbed, and the desorption temperature is monitored. Please note that the surface layers shown in the images are not a precise representation of species present on the surface but rather indicate an example of the anticipated outcome of the crosslinking procedure (see Discussion).

This study aims at comparing deposits prepared by electron irradiation from *n*-pentane, 2M2B, and (EtCp)_2_Ru with respect to their water binding properties in their pristine state and after subsequent treatment with a combination of adsorbed NH_3_ and electron irradiation. All experiments were thus carried out in an analogous fashion with all three precursors. The experimental sequence is summarized in Figure 3. Steps (a) to (i) represent all of the principal steps of the procedure as also introduced in our previous study on N incorporation into FEBID deposits made from (EtCp)_2_Ru [10].

In brief, vapors of a specific precursor were condensed onto the liquid nitrogen cooled Ta sheet held at temperatures between 100 K and 110 K. The intact precursor multilayers were subsequently exposed to an electron beam at an incident electron energy of E_0_ = 31 eV to induce crosslinking between the molecules leading to formation of a carbonaceous deposit (Figure 3a). After an electron exposure of 40 mC/cm^2^, remaining volatile irradiation products that stayed on the surface at 110 K were removed by annealing the surface to 450 K (Figure 3b). This overall step mimics a FEBID process although FEBID is typically performed at room temperature. The thermal chemistry that is thus involved in FEBID proceeds here during the annealing. The composition of the annealed deposits was subsequently monitored by AES at room temperature to confirm that a carbonaceous deposit was obtained in all cases (Figure 3c).

Deposit fabrication was followed by electron beam treatment in presence of NH_3_ in order to introduce N into the deposits. Adlayers of NH_3_ were prepared on the deposits again held at 100 K to 110 K by dosing an amount of vapor that corresponded to a pressure drop of 4 mTorr in the gas handling manifold. As deduced earlier, this yields a coverage in the monolayer regime on the Ta substrate [13] (Figure 3d). To induce chemical reactions that trigger bond formation between the carbonaceous deposit and the NH_3_ adlayer, we employed the same electron irradiation (E_0_ = 31 eV, 40 mC/cm^2^) and annealing procedure (up to 450 K) as for deposit formation (Figure 3e). To confirm N incorporation, AES was performed after the substrate had warmed up back to room temperature (Figure 3f). The sequence consisting of NH_3_ condensation, electron irradiation and annealing, referred to as a treatment ‘cycle’ in the following, was repeated several times in order to increase the amount of N incorporated into the deposits.

After several cycles of combined NH_3_ and electron treatment, the water binding properties of the modified deposits were assessed using TDS of sub-monolayers of H_2_O condensed onto of the deposits held at 100 K to110 K (Figure 3g) so that the H_2_O molecules adsorbed on the deposit surface (Figure 3h). In a final step, the desorption temperature of H_2_O was determined in a TDS run to quantify the water binding capabilities of the deposit (Figure 3i).

### 3.2. Preparation and Characterization of Carbonaceous Deposits

The aim of the experiments presented herein is to demonstrate how the binding of H_2_O depends on the incorporation of N in the carbonaceous deposits. Therefore, the deposits must be thick enough to rule out that AES includes contributions of N that may become bound to the underlying Ta substrate. To this end, the amount of precursor gas adsorbed on the substrate was adjusted so that subsequent electron irradiation yielded crosslinked deposits of sufficient thickness to screen signals from the underlying Ta substrate in AES (Ta MNN at 175 eV and 183 eV) [17]. In the case of (EtCp)_2_Ru, it was previously shown that an amount of vapor corresponding to a pressure drop of 2 mTorr in the gas inlet is sufficient to produce a deposit that quantitatively suppresses the Ta signal [10]. Therefore, the same vapor dose was used here to produce deposits used for incorporation of N (see Section 3.3). Based on the previous monolayer calibration for (EtCp)_2_Ru [12,13], this corresponds to an adsorbed precursor layer with a thickness of 8 ML. In the case of 2M2B, deposits were produced from increasing amounts of vapor to identify a thickness at which the Ta signals recorded after the electron irradiation step had fallen below the noise level (see Supporting Information, Figure S3). Based on these data, a vapor dose of 20 mTorr corresponding to 47 monolayers was selected for 2M2B. The same amount of vapor was also used in the case of *n*-pentane, corresponding to an adsorbate thickness of 69 ML prior to electron irradiation.

**Figure 4:**
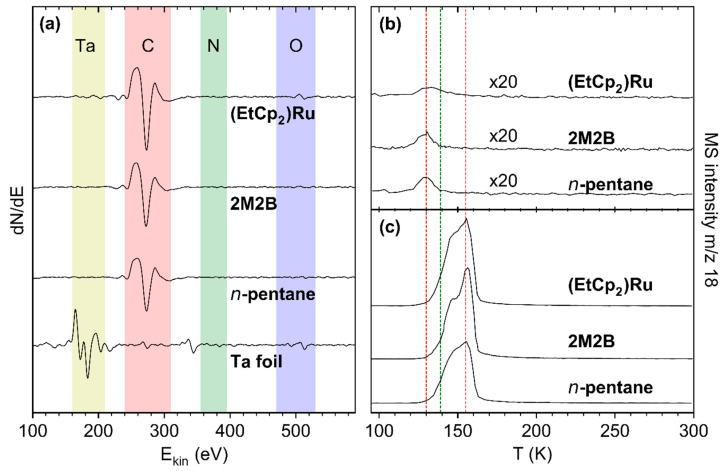
(**a**) AES of the freshly sputtered Ta substrate (bottom) and of deposits prepared from a 69 ML adsorbate of *n*-pentane, 47 ML of 2M2B, and 8 ML of (EtCp)_2_Ru by applying an electron exposure of 40 mC/cm^2^ at E_0_ = 31 eV (second from bottom to top). (**b**,**c**) TDS of H_2_O on the deposits shown in (**a**). The adsorbates were prepared by dosing an amount of H_2_O vapor corresponding to a pressure drop of (**b**) 0.08 mTorr and (**c**) 4 mTorr on the deposits. The red lines mark the sub-monolayer and multilayer desorption temperatures from the deposits while the green line represents the sub-monolayer desorption temperature of H_2_O on a rough carbon surface as reported previously [7].

For all deposits that were produced from the adsorbed precursor layers by electron irradiation as described in Section 3.1, AES data are in fact dominated by a signal at a kinetic energy of ~275 eV which is assigned to carbon (C KLL) [17] (Figure 4a). In the case of the (EtCp)_2_Ru deposit, however, the Ru MNN signal at 277 eV likely contributes to the intensity. Note that our instrument does not allow us to resolve the C KLL and Ru MNN features resulting in a single signal. We also note that a weak residual Ta signal is observed for (EtCp)_2_Ru. However, we will show in Section 3.3 that Ta does not contribute noticeably to the signals resulting from incorporation of N. Therefore, we consider the chosen deposit thickness to be sufficient for all three precursors.

The significantly larger adsorbate thickness needed in the case of the metal-free precursors to produce a deposit that efficiently screens AES signals from the underlying Ta substrate can relate to (i) the known longer attenuation length (AL) of electrons in absence of Ru [18] and (ii) a lower crosslinking efficiency. The AL for pure C is roughly twice as large than for pure Ru at the energy of the Ta MNN electrons. However, according to a previous estimate, deposits prepared from (EtCp)_2_Ru are rich in carbon with a composition between RuC_9_ and RuC_14_ [12,13]. Therefore, the AL for such deposits should be closer to that for pure C than to the value for pure Ru. This indicates that crosslinking reactions as needed to form a non-volatile deposit are more efficient in the case of (EtCp)_2_Ru than for 2M2B. The underlying chemistry will be discussed in Section 4.

Due to the intended screening of the Ta signal, the deposit thickness cannot be determined based on its attenuation. However, we can estimate a minimum deposit thickness based on the absence of the Ta signal. Considering the minimum intensity of the Ta signal that can be confidently distinguished from background noise as 2–3% of the signal from the sputter-cleaned Ta substrate in the individual deposit formation experiments and using tabulated electron AL values [18] interpolated to the kinetic energy of 183 eV for Auger electrons emitted from Ta, we estimate this minimum deposit thickness to be 2.5 nm for 2M2B, 2.4 nm for *n*-pentane, and 1.2–2.2 nm for (EtCp)_2_Ru. Note that for *n*-pentane and 2M2B, AL values for carbon were used, while in the case of (EtCp)_2_Ru, a composite material with unknown precise elemental composition must be assumed. We therefore estimate a thickness range based on the AL in both C and Ru, respectively, and expect the minimum deposit thickness to fall into this range. To put these values in perspective, we note that the diameter of a molecule of the precursor MeCpPtMe_3_ has been reported as 0.93 nm [16]. This value also provides an order of magnitude of the size of (EtCp)_2_Ru. A thickness pf 1.2–2.2 nm would thus correspond to a thickness of around 2 ML of intact precursor. This is much less than the thickness of the initial precursor adsorbate of about 8 ML. However, the small residual Ta signal indicates that, even for (EtCp)_2_Ru, the thickness is noticeably reduced after electron irradiation and annealing to remove remaining precursor molecules in line with previous results obtained by RAIRS [10]. Overall, this points to an incomplete retention of the precursor material in the deposit which is, however, more pronounced in the case of the metal-free compounds 2M2B and *n*-pentane.

During conversion of the adsorbed layers to the crosslinked carbonaceous deposit, volatile neutral compounds evolving from the surface were monitored to reveal fragmentation of the precursors. ESD of small hydrocarbons is evident for all three precursors from mass spectra recorded during irradiation (see Supporting Information, Figures S4 and S5 for *n*-pentane and 2M2B, and previous results [10] for a detailed discussion of the electron-induced decomposition of (EtCp)_2_Ru). TDS data recorded during the annealing step following electron irradiation of *n*-pentane and 2M2B can be found in Supporting Information, Figures S6 and S7 and as previously reported [12] for (EtCp)_2_Ru. Similar to ESD, desorption at *m*/*z* 27, 29 and 39, 41 was seen within a temperature range of 120–150 K. In contrast, small signals at *m*/*z* 70 (M^•+^ of 2M2B) and *m*/*z* 72 (M^•+^ of *n*-pentane), respectively, point to desorption of unreacted precursor molecules. The identity of the desorbing species was confirmed by MS acquired upon dosing of the precursors to the vacuum chamber (see top spectra in Supporting Information, Figures S6 and S7). Deposit formation was also monitored by RAIRS (see Supporting Information, Figure S8 for 2M2B and as previously reported [10] for (EtCp)_2_Ru)). In the case of 2M2B, electron irradiation leaves behind some intensity on the characteristic initial C=CH and CH_3_ bending vibration bands (1300–1500 cm^−^^1^) [19,20]. After annealing and consequent removal of residual intact 2M2B from the deposit, the former has disappeared leaving only the signatures of the CH_3_ bending vibrations behind. We also note that, in line with the previous results for (EtCp)_2_Ru [10], some remaining intensity in the range of the CH stretching vibrations (2800–3000 cm^−^^1^) reveals the persistent hydrogen content of the deposit. This is relevant for the chemistry that underlies the subsequent incorporation of N as described in the following Sections.

To characterize the water-binding properties of the deposits, small quantities of H_2_O amounting to sub-monolayer coverage were condensed onto the deposit surface. As the maximum of the H_2_O desorption peak monitored at *m*/*z* 18 continuously shifted towards the multilayer desorption temperature of 155 K with increasing surface coverage (see Supporting Information, Figures S9–S11), a vapor dose leading to a full monolayer cannot be derived by deconvolution of the desorption signal into components for desorption from the multi- and monolayer. We therefore chose a surface coverage corresponding to a pressure drop of 0.08 mTorr in the gas handling manifold that produced a H_2_O desorption signal peaking at 130.3 ± 1.7 K for all three deposits (Figure 4b). This desorption temperature is even lower than the temperature of 139 K observed for desorption of sub-monolayer amounts of H_2_O from a rough carbon surface [7] and thus also indicative of a sub-monolayer coverage on the deposits studied here. Such a low desorption temperature reveals that the interactions between the polar adsorbate H_2_O and the deposit are very weak indicative of a non-polar surface. It also rationalizes the low sensitivity reported for humidity sensing based on carbonaceous deposits fabricated by FEBID [4]. We note for comparison that, for all deposits in this study, H_2_O multilayer desorption was observed between 150–160 K (Figure 4c and Supporting Information, Figures S9–S11).

### 3.3. Incorporation of Nitrogen in the Deposits and Effect on Binding of H_2_O

**Figure 5:**
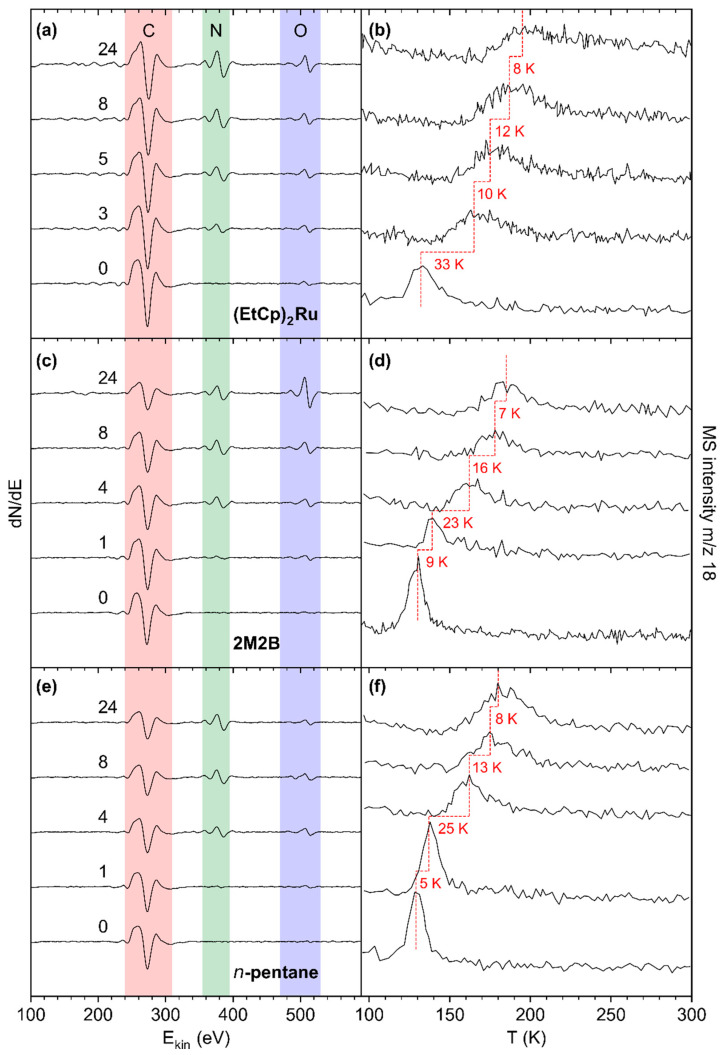
AES of pristine deposits prepared from (**a**) (EtCp)_2_Ru, (**c**) 2M2B, and (**e**) *n*-pentane (bottom spectrum in each panel) and of the same deposits after increasing number of treatment cycles by electron irradiation (40 mC/cm^2^ at E_0_ = 31 eV) in presence of NH_3_ (4 mTorr). Respective TDS acquired at *m*/*z* 18 (H_2_O^•+^) after the same treatment cycles and subsequent dosing of H_2_O (0.08 mTorr) onto the deposits prepared from (**b**) (EtCp)_2_Ru, (**d**) 2M2B, and (**f**) *n*-pentane. The dashed red lines serve as a guide to the eye to facilitate observing shifts of the H_2_O desorption signal. Temperature shifts between the presented cycles are denoted in red next to the respective TD spectra.

Deposit modification via combined NH_3_ and electron treatment was carried out for all pristine deposits, as described in Section 3.1. After condensing thin adlayers of NH_3_ onto the deposit surface held at T = 110 K, electron exposure at E_0_ = 31 eV was resumed. An increase in the *m*/*z* 28 signal above the background level in ESD pointed to formation and desorption of molecular nitrogen (N_2_) resulting from electron-induced decomposition of NH_3_ (see Supporting Information, Figure S12) in accordance with previous experiments for (EtCp)_2_Ru [10]. AES data give evidence that N was also incorporated in the deposit (Figure 5, left row of panels). TDS data reveal the effect of this modification on the desorption temperature T_DES_(H_2_O) of sub-monolayer quantities of H_2_O from the deposits (Figure 5, right row of panels). Note that Figure 5 displays only selected representative cycles of treatment. Complete data sets are compiled in Figures S13–S15 of the Supporting Information.

The effect of combined NH_3_ and electron treatment was first assessed after the first cycle for the deposits produced from 2M2B and *n*-pentane and after three cycles in the case of (EtCp)_2_Ru. In the latter case, in addition to the C KLL signal, a new signal at ~386 eV assigned to the N KLL transition became clearly visible (Figure 5a). This signal was still barely detectable for the *n*-pentane and 2M2B deposits after the first cycle but also well visible after four cycles (Figure 5c,e). For all deposits, the N KLL signal had further increased after eight cycles while a trend towards saturation can be noted from the AES data acquired after 24 cycles.

The N KLL signal points to successful incorporation of N into the deposits. This is supported by an enlarged plot of the N KLL transition (see Supporting Information, Figure S16). The N KLL signal serves as a probe for the chemical state in which N is bound. For instance, the peak shapes of metallic nitrides can be clearly distinguished from those of covalently bound N-containing species [21]. In line with our previous argumentation [10], the wide spacing of features within the N KLL transition of all treated deposits indicates the formation of a material with similar bonding as in covalent compounds with C-N bonds [21]. A control experiment that applied electron irradiation in presence of NH_3_ to the bare Ta substrate confirmed the distinctly different shape of the N KLL band for N bound to Ta (Supporting Information, Figure S16). This gives clear evidence that N is incorporated in the carbonaceous deposit during the treatment cycles.

The incorporation of N is also reflected by desorption of sub-monolayer amounts of H_2_O as monitored by TDS (Figure 5b,d,f, see Supporting Information, Figures S17–S19 for TDS results obtained for varying H_2_O adsorbate thickness). For all deposits, T_DES_(H_2_O) shifted towards higher temperatures with the largest shift observed for the (EtCp)_2_Ru deposit (+33 K after three cycles as compared to +32 K for 2M2B and +30 K for *n*-pentane after four cycles, respectively). The trends continued for all three deposits up to the maximum cycle number of 24 but with decreasingly strong shifts of T_DES_(H_2_O), i.e., a saturation desorption temperature for each deposit was successively approached.

At first sight, the maximum shift of T_DES_(H_2_O) appears to correlate with the intensity of the N KLL AES signal which, after 24 cycles, is higher for the (EtCp)_2_Ru deposit (Figure 5a) as compared to the deposits prepared from 2M2B (Figure 5c) and *n*-pentane (Figure 5e). However, it is important to note that the C:N ratio derived from the relative intensities of the C KLL and N KLL signals is similar for all three deposits. This evaluation is based on the Peak-to-Peak (PtP) height of the signals corrected by the sensitivity factors [17] at 5 keV of 0.4763 for C and 0.9157 for N. This leads to C:N elemental ratios of 3.3:1 for *n*-pentane and 3.7:1 for 2M2B after 24 cycles of treatment. In the case of (EtCp)_2_Ru, the overlap of the Ru MNN signal with C KLL must be considered. According to a previous estimate, the deposit composition lies in the range between RuC_9_ and RuC_14_, leading to a contribution between 22% and 30% of the Ru MNN signal to the intensity of the overall signal [12,13]. After subtraction of this contribution, a C:N ratio between 2.9:1 and 3.2:1 was derived after 24 cycles (see Supporting Information, Tables S1–S3). In conclusion, relatively similar C:N ratios were obtained for all three precursors.

**Figure 6:**
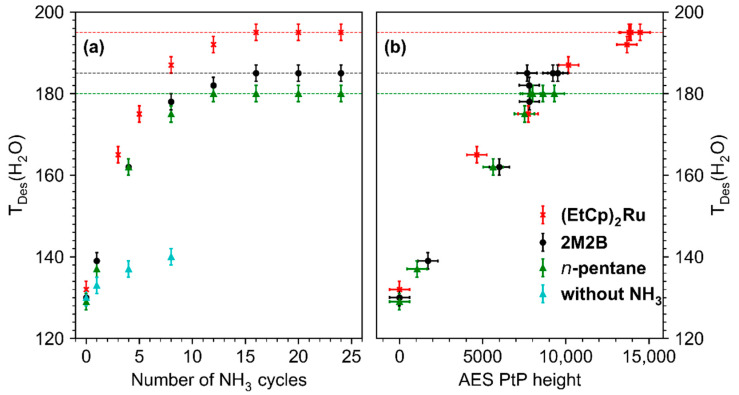
(**a**) Dependence of the H_2_O desorption temperature (T_DES_(H_2_O)) on the number of treatment cycles (4 mTorr NH_3_, 31 eV, 40 mC/cm^2^) carried out on deposits fabricated from *n*-pentane (green), 2M2B (black), (EtCp)_2_Ru (red), and 2M2B in absence of NH_3_ (blue, for details, see Supporting Information, Figure S20). Dashed horizontal lines mark the maximum T_DES_(H_2_O) that can be reached by repeated treatment cycles. Error bars were estimated based on the width of the desorption signals with the center representing the maximum of the desorption signal. (**b**) Dependence of the H_2_O desorption temperature (T_DES_(H_2_O)) on the Peak-to-Peak (PtP) height of the derivative N KLL signal in AES. The horizontal error bars for the PtP height were derived by considering the noise level in the derivative AES data.

The shift of T_DES_(H_2_O) is quantitatively summarized in Figure 6a which shows a plot of the maximum of T_DES_(H_2_O) against the number of treatment cycles for all studied deposits. For the pristine deposits, T_DES_(H_2_O) lies in the range 129–132 K, a temperature well below the multilayer desorption temperature of H_2_O (155 K, see Figure 4b and Figure S9–S11 of the Supporting Information). With ongoing treatment, T_DES_(H_2_O) increased for all deposits but shifted most strongly for the deposit produced from (EtCp)_2_Ru. After 10–15 cycles, T_DES_(H_2_O) converged toward a constant value for all deposits. This maximum shift is marked by dashed horizontal lines in Figure 6a. It is highest for deposits prepared from (EtCp)_2_Ru with a maximum T_DES_(H_2_O) of 195 ± 2 K, followed by 2M2B (185 ± 2 K) and *n*-pentane (180 ± 2 K). In addition, the shift of T_DES_(H_2_O) shows an approximately linear dependence on the intensity of the N KLL signal in AES for all three deposits (Figure 6b). This indicates that by varying the amount of N incorporated in the carbonaceous deposits, T_DES_(H_2_O) can be tuned in a predictable manner up to the maximum value that, however, depends on the chemical nature of the precursor used to prepare the deposit. This dependence on the molecular structure of the precursor will be further discussed in Section 4.

We note that for all three deposits, not only the N KLL signal but also the O KLL signal at a kinetic energy of ~510 eV [17] increased during the treatment. This signal can be traced back to oxidation reactions triggered by the electron-induced chemistry of residual H_2_O that is present in the background gas of the UHV chamber and can also adsorb on the deposit surface when cooled down to T = 110 K. To rule out that the incorporation of O leads to the observed shifts in T_DES_(H_2_O), we performed the same experimental sequence for a total number of eight cycles in the absence of NH_3_ on the example of a deposit produced from 2M2B (see Supporting Information, Figure S20). A similar degree of oxidation as observed in Figure 4 can be seen also in this experiment by a successive increase in the O KLL intensity in AES. In contrast to Figure 4, however, no significant shift of T_DES_(H_2_O) was observed indicating that the presence of NH_3_ is crucial to induce the desired surface modification. Rather, a continuous decrease in the intensity and broadening of the H_2_O desorption signal was observed, indicative of water-assisted removal of material [12,22] leading to formation of pores in the carbonaceous matrix in which H_2_O is retained for a prolonged time upon annealing.

## 4. Discussion

The results shown in Figure 6 reveal that incorporation of nitrogen during electron irradiation in presence of NH_3_ can be used to tune the binding strength of H_2_O in the deposit. The shift of the desorption temperature of H_2_O upon nitrogen incorporation is most pronounced in the case of the deposit prepared from (EtCp)_2_Ru and less so for 2M2B and *n*-pentane. The effect thus depends on the chemical nature of the precursors. As shown in Section 3.2, a different chemical behavior was also noted during deposit formation where the irradiated material obtained from (EtCp)_2_Ru was more efficiently retained on the surface than in the case of the olefin 2M2B. Therefore, we discuss first chemical reactions that contribute to the formation of the non-volatile deposit before considering those that lead to the uptake of nitrogen in the deposit. The overall chemistry of these processes is complex and leads to a variety of different structures. Therefore, a detailed molecular structure of the deposit cannot be deduced from the data presented here. However, depending on the precursor architecture, different reactions are anticipated. Based on mechanistic insight brought forward in different earlier studies which we review in the following, we attempt to rationalize the results obtained herein. We note that a more comprehensive discussion of the reactions involved in nitrogen uptake during electron irradiation in presence of NH_3_ has been brought forward previously for the case of (EtCp)_2_Ru [10].

**Figure 7:**
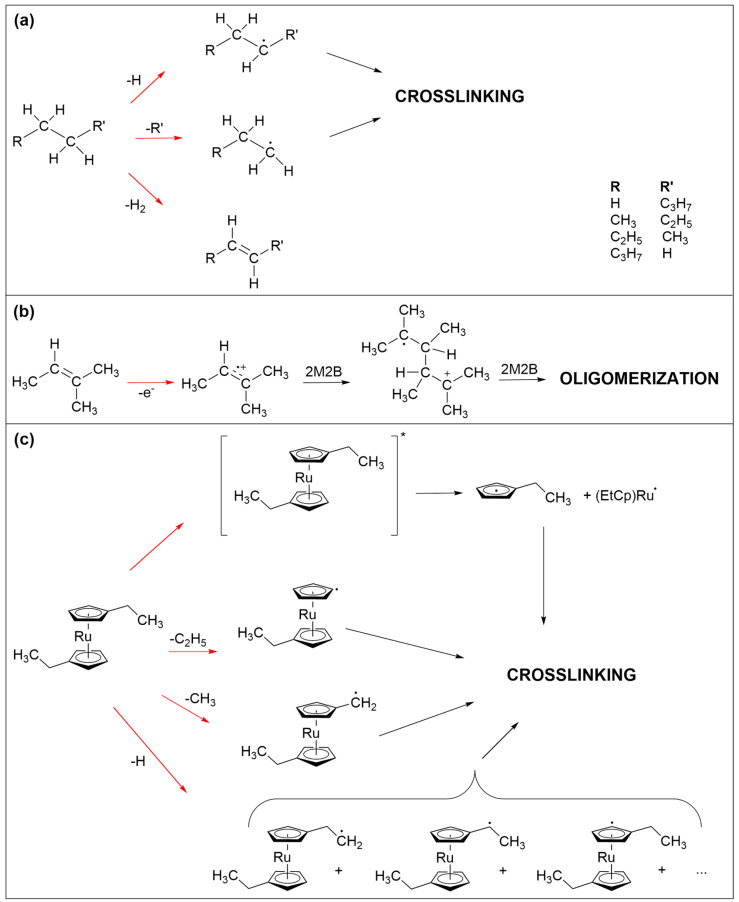
Electron-induced processes that are held to be involved in the crosslinking reactions that lead to the formation of the non-volatile carbonaceous deposit from the three precursors studied herein. (**a**) C-H and C-C bond cleavage in saturated hydrocarbons represented herein by *n*-pentane. The resulting radicals can recombine to crosslink the fragments. (**b**) Oligomerization initiated by electron ionization of olefins represented herein by 2M2B. The radical cation can add to an adjacent double bond to yield a larger radical cation which can carry on as a chain reaction. In addition, C-H and C-C bond cleavage is also anticipated here. (**c**) Neutral dissociation following electronic excitation as well as different C-H and C-C bond cleavages in (EtCp)_2_Ru. The resulting radicals can again recombine to crosslink the fragments. In all frames, red arrows imply electron-induced reactions while black arrows denote subsequent thermal reactions. For simplicity, electron-induced fragmentation processes (DEA, ND, DI) are not depicted in detail but the reaction schemes focus on those radical species that are relevant for crosslinking within the precursor layer.

The crosslinking reactions that are most likely involved in the formation of the non-volatile carbonaceous deposit from the three precursors studied herein are schematically summarized in Figure 7. At the electron energy applied in the presents study, different electron–molecule interactions are accessible [23]. The incident electron can trigger ionization of the precursor molecules both without (electron ionization, EI) or with subsequent dissociation (dissociative ionization, DI) as well as electronic excitation followed by dissociation (neutral dissociation, ND). Electrons that are slowed down by inelastic scattering or secondary electrons released in an ionization event can further have a sufficiently low energy to attach to a precursor and thereby induce fragmentation (dissociative electron attachment, DEA). For simplicity, we do not consider these processes in detail but focus in particular on neutral radical fragments which can undergo recombination with a second radical species leading to crosslinking within the precursor layer.

In the case of saturated hydrocarbons, represented herein by *n*-pentane (Figure 7a), electron irradiation typically leads to cleavage of C-H or C-C bonds as well as loss of H_2_ [24,25,26]. The latter yields products with double bonds [25,26]. Bond cleavage leads to smaller and more volatile fragments of which at least one is a neutral radical. Radicals can recombine but small fragments can also desorb [24], removing material from the deposit. The latter is also evident from the ESD results showing desorption of hydrocarbon fragments with up to three carbon atoms (Supporting Information, Figure S4). Considering furthermore that small radical species are more mobile than larger ones, crosslinking should yield not only non-volatile material but also a substantial amount of relatively small products that can desorb during TDS and annealing.

Similar bond cleavage reactions upon electron irradiation are also anticipated for 2M2B as again supported by ESD data (Supporting Information, Figure S5). Here, desorption of species with three carbon atoms is less pronounced than in the case of *n*-pentane. This is not unexpected because olefins can also oligomerize following electron impact ionization [23] as shown in Figure 7b. Note that this type of reaction can also proceed in adsorbates of *n*-pentane as soon as a sufficient number of double bonds have been formed by loss of H_2_. Ionization removes an electron from the CC double bond and the resulting radical cation can add to the double bonds of a nearby neutral olefin to form the radical cation of the dimer. This can, in principle, carry on as a chain reaction that would produce large and less volatile products. However, the low efficiency of deposit formation noted for 2M2B suggests that this reaction is at least partially impeded by the bulky side groups attached to the double bond. This together with the bond cleavage reactions that will also occur in product oligomers rationalizes the low efficiency of deposit formation from 2M2B as compared to (EtCp)_2_Ru.

Bond cleavage upon electron irradiation is less pronounced in (EtCp)_2_Ru. As discussed previously [12,22], the ethyl side chains are preferably dissociated in this compound (Figure 7c). In contrast, and as discussed more comprehensively for the precursor MeCpPtMe_3_ [16,27], the cyclopentadienyl rings are difficult to remove from the deposit. In the pristine state, the anionic Cp ligand is an aromatic system and thus particularly stable. Electronic excitation of cyclopentadienyl complexes can, however, dissociate a neutral cyclopentadienyl with radical centered on the ring from the metal [28]. In fact, the formation of metal nanoparticles in UV photolysis experiments has been described previously in the case of ferrocene (Cp_2_Fe) [29,30]. An analogous neutral dissociation process induced by electron-induced excitation of (EtCp)_2_Ru (see Figure 7c, top) and other cyclopentadienyl complexes is a plausible explanation for the formation of the metal particles and thus of the nanogranular deposits as observed in FEBID [3,4,12]. The cyclopentadienyl radical is considered as an important intermediate in the formation of larger products such as polycyclic aromatic hydrocarbons [31,32,33]. It is thus possible that similar reactions contribute to the formation of the non-volatile carbonaceous matrix in FEBID when cyclopentadienyl-containing precursors are applied. As an alternative reaction pathway and in analogy to the efficient crosslinking known from aromatic self-assembled monolayers (SAMs) [25], radical sites resulting from C-H bond cleavage on the Cp ring can recombine which, due to the less extensive fragmentation and in contrast to *n*-pentane and 2M2B, yields significantly larger and less volatile species. This rationalizes that, in the case of (EtCp)_2_Ru, a thinner adsorbate is sufficient to produce a deposit that fully screens the AES signals from the underlying Ta substrate.

Overall, this literature review also suggests that the carbonaceous deposits produced in the present study by electron irradiation of (EtCp)_2_Ru contain crosslinked aromatic ring structures. In the case of aromatic SAMs, such crosslinking was recently shown to be accompanied by formation of nanosized voids [34]. Considering this, the chemical structure of the deposit resulting from (EtCp)_2_Ru is most likely different than that of deposits made from *n*-pentane and 2M2B which are built up from short and more flexible hydrocarbon chain fragments. Nonetheless, the desorption temperature of H_2_O is the same for all three deposits prior to incorporation of N which indicates that all three pristine deposits have a comparable hydrophobicity.

**Figure 8:**
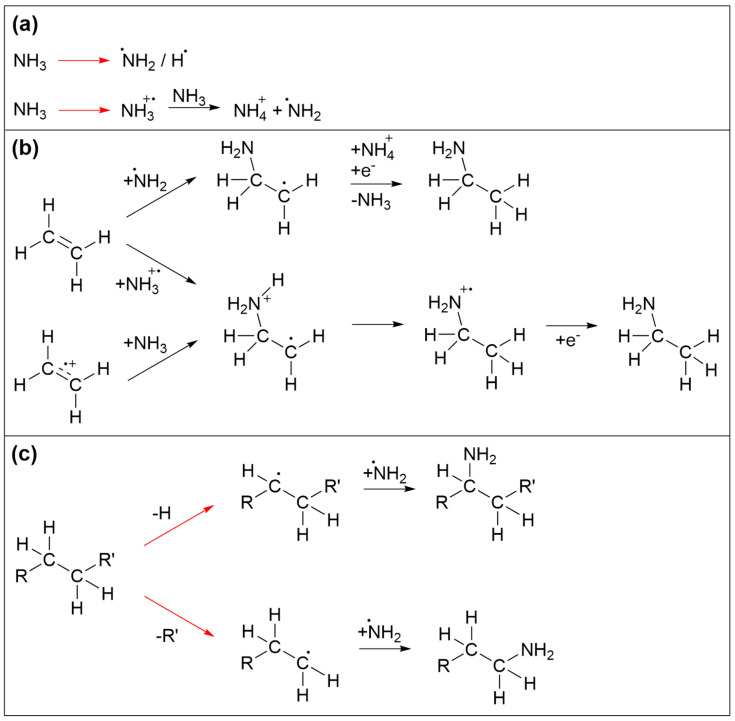
Prototypical reactions that lead to incorporation of N in carbonaceous deposits during electron irradiation in presence of NH_3_. (**a**) Activation of NH_3_ by electron-induced fragmentation yielding radical fragments. Electron ionization in presence of further NH_3_ triggers proton transfer as an alternative pathway to an NH_2_ radical. (**b**) Reactions of olefins with activated NH_3_ [35]. After ionization, either the NH_2_ radical or the radical cation of NH_3_ can add to a C=C double bond. In the first case (top) neutralization by a thermal electron triggers transfer of an H from NH_4_^+^ to the adduct yielding the neutral aminated product. In the second case, intramolecular H migration and capture of a thermalized electron yield the product. Alternatively, this latter reaction sequence can also be initiated by electron ionization of the olefin. (**c**) Recombination of hydrocarbon radicals with NH_2_ radicals. In all frames, red arrows imply electron-induced reactions while black arrows denote subsequent thermal reactions. For simplicity, electron-induced fragmentation processes (DEA, ND, DI) are not depicted in detail, but the reaction schemes focus on those radical species that are relevant for formation of C-NH_2_ bonds. Note that further reactions of other intermediates such as NH or atomic N that may also result from dissociation of NH_3_ as well as subsequent chemistry of aminated products are omitted for simplicity.

The reaction sequences that trigger incorporation of N in the deposits are again initiated by electron irradiation. Prototypical reactions are summarized in Figure 8. Again, we focus mainly on the essential reaction products without considering in detail the specific electron-molecule interactions. Among the species produced by electron interaction with NH_3_, NH_2_ radicals are of particular relevance (Figure 8a). Electron-induced reactions of NH_3_ with the ethylene (C_2_H_4_) have been discussed in depth previously [35,36,37]. It was shown that electrons with sufficient energy can induce addition of NH_3_ to the double bond of C_2_H_4_ to yield ethylamine. While this was initially thought to be triggered by direct addition of an ionized reactant to the neutral second reactant [37], a theoretical study revealed that a more favorable reaction pathway involved first proton transfer from an ionized to a neutral NH_3_ (Figure 8a). This yields stable NH_4_^+^ and a NH_2_ radical that can add to one of the carbon atoms involved in the C=C double bond while a hydrogen atom is transferred to the other carbon atom upon neutralization of the reaction complex by a thermalized electron (Figure 8b) [35]. In addition, NH_2_ can also be formed by DEA to NH_3_ [38] or by ND of NH_3_ [35]. NH_2_ can also recombine with radical species produced by electron-induced bond cleavage of saturated hydrocarbon molecular units (Figure 8c) Note that, in the case of a radical produced by C-H cleavage, recombination with NH_2_ substitutes a hydrogen atom of an intact molecular unit (Figure 8c). NH_2_ can also react with intact hydrocarbons. However, as shown previously for several prototypical alkylbenzenes [39], i.e., molecules containing both an aromatic ring and a saturated side chain akin to the EtCp ligand, abstraction of H from the side chain and in particular from the position adjacent to the aromatic ring is favored over addition of NH_2_ to the ring. In contrast, substitution of an H atom in benzene by NH_2_ has been demonstrated, for instance, in a photocatalytic process that proceeds in an aqueous solution of NH_3_ in presence of the catalyst material consisting of Pt nanoparticles supported by TiO_2_ [40]. In this process, formation of an electron–hole pair assists the release of the NH_2_ radical and the surface-mediated production of H_2_ resulting from recombination of the H atoms cleaved from NH_3_ and benzene. In the present electron-driven process, NH_2_ radicals are produced by electron impact, and it is possible that additional radical species present in the vicinity assist the removal of an H at a site where the NH_2_ binds to an aromatic ring. However, it is difficult to estimate the contribution of such a reaction to the incorporation of N in the deposits investigated herein. We also note that, as proposed earlier [10], EtCp ligands which remain attached to Ru after deposit formation may also be dissociated from the metal by protonation which converts the ligand to neutral ethylcyclopentadiene and thereby weakens interaction with the metal. Protonation is possible by transfer of H^+^ from an ionized NH_3_^+^ to an EtCp ligand instead of a second NH_3_ as shown in Figure 8a. As an olefin, neutral ethylcyclopentadiene may again involve in reactions as shown in Figure 8b which lead to addition of NH_3_ to a C=C double bond. Overall, the carbonaceous deposit produced from (EtCp)_2_Ru is thus probably sufficiently disordered to still retain a certain amount of saturated hydrocarbon groups or double bonds that are not implicated in an aromatic ring. In consequence, NH_2_ radicals can react with different molecular sites to initiate the incorporation of N in the deposit.

The distinctly different T_DES_(H_2_O) observed after 24 cycles of treatment by electron irradiation in presence of NH_3_ suggest that despite the comparable H_2_O adsorption on the pristine deposits, incorporation of N has a stronger effect on the H_2_O binding strength in the case of (EtCp)_2_Ru. However, for all three deposits, incorporation of nitrogen does not simply lead to decay of the initial low-temperature desorption signal and a concomitant appearance of a new signal at higher temperature as would be anticipated if one type of binding site was replaced by a new one. Instead, the desorption signal shifts continuously towards higher temperature (Figure 5). In line with this and as obvious from Figure 6b, T_DES_(H_2_O) from the deposits increases linearly with the intensity of the N KLL AES signal. Furthermore, the C:N elemental ratio achieved after 24 treatment cycles is similar for all three deposits suggesting that the different electron-induced reactions that bind N to the deposit are of comparable efficiency. The observed differences between the maximum shift of T_DES_(H_2_O) thus cannot be traced back to a different amount of N. Furthermore, the continuous shift of T_DES_(H_2_O) indicates that the binding of H_2_O to the deposit is not dictated by localized binding sites but that incorporation of nitrogen increases the overall interaction of the carbonaceous deposit with the adsorbate. Considering the anticipated formation of voids in deposits prepared from (EtCp)_2_Ru, these structures may provide particularly strong binding sites after incorporation of N because of the combined effect of the functional groups and a nanoscale porosity. However, a more detailed analysis of this effect is beyond the scope of this work.

## 5. Conclusions

With this study, we provide evidence that the binding strength of H_2_O to carbonaceous deposits prepared by electron irradiation of adsorbed volatile precursor molecules can be tuned by incorporating nitrogen in the deposits. Such carbonaceous deposits are typically obtained in FEBID processes that employ metal organic precursors with large organic ligands as exemplified herein by (EtCp)_2_Ru. For comparison, deposits were also prepared from the saturated hydrocarbon *n*-pentane and the olefin 2M2B. Incorporation of nitrogen was achieved under UHV conditions by condensing NH_3_ on top of the deposit surface held at cryogenic temperature and subsequently exposing the adsorbate to electron irradiation. However, as demonstrated previously [10], the same deposit modification can also be performed by electron irradiation in presence of an NH_3_ atmosphere in a scanning electron microscope used for the actual FEBID process. In both cases, the amount of N incorporated into the deposits can be tuned by adjusting the duration of treatment.

In the present study, we have shown that nitrogen incorporation leads to an increased binding of H_2_O to the deposit. This was revealed by TDS experiments performed with sub-monolayer quantities of H_2_O that were adsorbed on the modified surfaces. With ongoing treatment time, the desorption temperature successively shifted towards higher values in line with stronger adsorption which presumedly results from the ability of nitrogen-containing groups to involve in hydrogen bonds. While similar quantities of N were incorporated in all of the deposits prepared from the three different precursor compounds, the best performance with respect to tunability was observed for (EtCp)_2_Ru. This result is tentatively ascribed to the nature of the carbonaceous deposit which, in the case of (EtCp)_2_Ru is likely to contain rigid aromatic ring structures and nanoscale voids while crosslinking of the small and more mobile fragments resulting from electron-induced fragmentation of *n*-pentane and 2M2B probably leads to a less porous deposit.

In summary, this proof-of-concept study confirms enhanced water-binding properties of deposits fabricated by electron exposure when an additional treatment step involving NH_3_ and electron irradiation is conducted. This offers an approach to improve the sensitivity of humidity sensors based on carbonaceous deposits fabricated by FEBID. As a next step, the charge transport in the N-doped deposits needs to be investigated. However, as previously shown by electron microscopy [10], the shape of the deposit deteriorates during this procedure. This was traced back to bubble formation by gaseous electron-induced decomposition products of NH_3_ (mainly N_2_ and H_2_) [41]. Upcoming studies will therefore focus on the implementation of high concentrations of hydrogen bond donor/acceptor species such as -NH_2_ groups without employing post-deposition treatment with NH_3_. Precursor molecules containing NH_2_ or NH_3_ ligands often possess low vapor pressures [41,42]. With the method of ion soft-landing, however, such low volatility compounds (also including the class of ionic compounds that are not yet available for FEBID) can be brought into the gas phase to be deposited in smooth, rupture-free surface layers [43]. Such layers could be subsequently crosslinked and patterned by use of a focused high-energy electron beam.

## Data Availability

Data presented in this study are available on request from the corresponding author.

## Supplementary Materials

The following supporting information can be downloaded at: https://www.mdpi.com/article/10.3390/nano12244455/s1, Figure S1: coverage-dependent TD spectra of *n*-pentane; Figure S2: coverage-dependent TD spectra of 2M2B; Figure S3: AES of deposits prepared from different amounts of 2M2B; Figure S4: ESD-MS for *n*-pentane; Figure S5: ESD-MS for 2M2B; Figure S6: MS of *n*-pentane during gas dosing and TDS; Figure S7: MS of 2M2B during gas dosing and TDS and TDS curves of characteristic *m*/*z*; Figure S8: RAIR spectra acquired from a pristine layer of 2M2B, after electron exposure and after TDS; Figure S9: TDS of H_2_O on a fresh deposit produced from *n*-pentane; Figure S10: TDS of H_2_O on a fresh deposit produced from 2M2B; Figure S11: TDS of H_2_O on a fresh deposit produced from (EtCp)_2_Ru; Figure S12: ESD-MS for NH_3_ condensed on top of deposits produced from *n*-pentane, 2M2B and (EtCp)_2_Ru; Figure S13: (a) AES of pristine deposits prepared from n-pentane (bottom spectrum in each panel) and of the same deposits after increasing number of treatment cycles by electron irradiation (40 mC/cm^2^ at E_0_ = 31 eV) in presence of NH_3_ (4 mTorr). (b) Respective TDS acquired at *m*/*z* 18 (H_2_O^•+^) after the same treatment cycles and subsequent dosing of H_2_O (0.08 mTorr) onto the deposits prepared from n-pentane. The dashed red lines serve as a guide to the eye to facilitate observing shifts of the H_2_O desorption signal. Temperature shifts between the presented cycles are denoted in red next to the respective TD spectra; Figure S14: (a) AES of pristine deposits prepared from 2M2B (bottom spectrum in each panel) and of the same deposits after increasing number of treatment cycles by electron irradiation (40 mC/cm^2^ at E0 = 31 eV) in presence of NH_3_ (4 mTorr). (b) Respective TDS acquired at *m*/*z* 18 (H_2_O^•+^) after the same treatment cycles and subsequent dosing of H_2_O (0.08 mTorr) onto the deposits prepared from 2M2B. The dashed red lines serve as a guide to the eye to facilitate observing shifts of the H_2_O desorption signal. Temperature shifts between the presented cycles are denoted in red next to the respective TD spectra; Figure S15: (a) AES of pristine deposits prepared from (EtCp)_2_Ru (bottom spectrum in each panel) and of the same deposits after increasing number of treatment cycles by electron irradiation (40 mC/cm^2^ at E0 = 31 eV) in presence of NH_3_ (4 mTorr). (b) Respective TDS acquired at *m*/*z* 18 (H_2_O^•+^) after the same treatment cycles and subsequent dosing of H_2_O (0.08 mTorr) onto the deposits prepared from (EtCp)_2_Ru. The dashed red lines serve as a guide to the eye to facilitate observing shifts of the H_2_O desorption signal. Temperature shifts between the presented cycles are denoted in red next to the respective TD spectra; Figure S16: Enlarged AES of the N KLL transition; Figure S17: TDS of H_2_O on a fully processed deposit produced from *n*-pentane; Figure S18: TDS of H_2_O on a fully processed deposit produced from 2M2B; Figure S19: TDS of H_2_O on a fully processed deposit produced from (EtCp)_2_Ru; Table S1: Evaluation of the C:N ratio in a deposit produced from *n*-pentane; Table S2: Evaluation of the C:N ratio in a deposit produced from 2M2B; Table S3: Evaluation of the C:N ratio in a deposit produced from (EtCp)_2_Ru; Figure S20: AES and TDS data on electron treatment of a deposit produced from 2M2B. References [12,17] are cited in supplementary materials.
