# Supplementary material for: Towards Improved Humidity Sensing Nanomaterials via Combined Electron and NH3 Treatment of Carbon-Rich FEBID Deposits
Source: Nanomaterials (Basel). 2022 Dec 15;12(24):4455. doi: 10.3390/nano12244455 (PMC9785463; doi:10.3390/nano12244455)
Supplement: Supplementary file 1 [file nanomaterials-12-04455-s001.zip › nanomaterials-2097557-supplementary.pdf]

Supporting Information

to

Towards Improved Humidity Sensing Nanomaterials via Combined Electron and NH_3 Treatment of Carbon-Rich FEBID Deposits

Hannah Boeckers [1], Petra Swiderek [1] and Markus Rohdenburg [1,2,*]

[1] Institute for Applied and Physical Chemistry, University of Bremen, Leobener Str. 5, 28359 Bremen, Germany

[2] Wilhelm-Ostwald-Institute for Physical and Theoretical Chemistry, Leipzig University, Linnéstr. 2, 04103 Leipzig, Germany

* Correspondence: markus.rohdenburg@uni-leipzig.de; Tel.: +49-341-9736505.

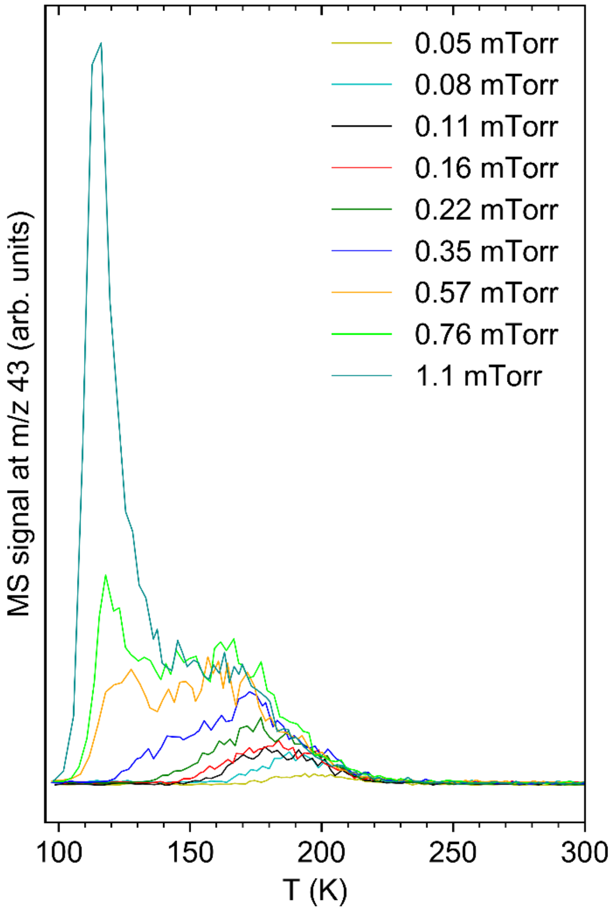

Figure S1. Thermal desorption spectra of thin layers of *n*-pentane recorded after dosing various amounts of vapor to the Ta sheet held at 110 K. m/z 43 ($C_3H_7^+$) was chosen as a representative fragment of *n*-pentane. The multilayer desorption signal with maximum at 115 K started to emerge when a vapor dose of 0.35 mTorr was leaked onto the substrate. This gives evidence that the monolayer was saturated when a vapor dose between 0.22 mTorr and 0.35 mTorr was leaked onto the substrate.

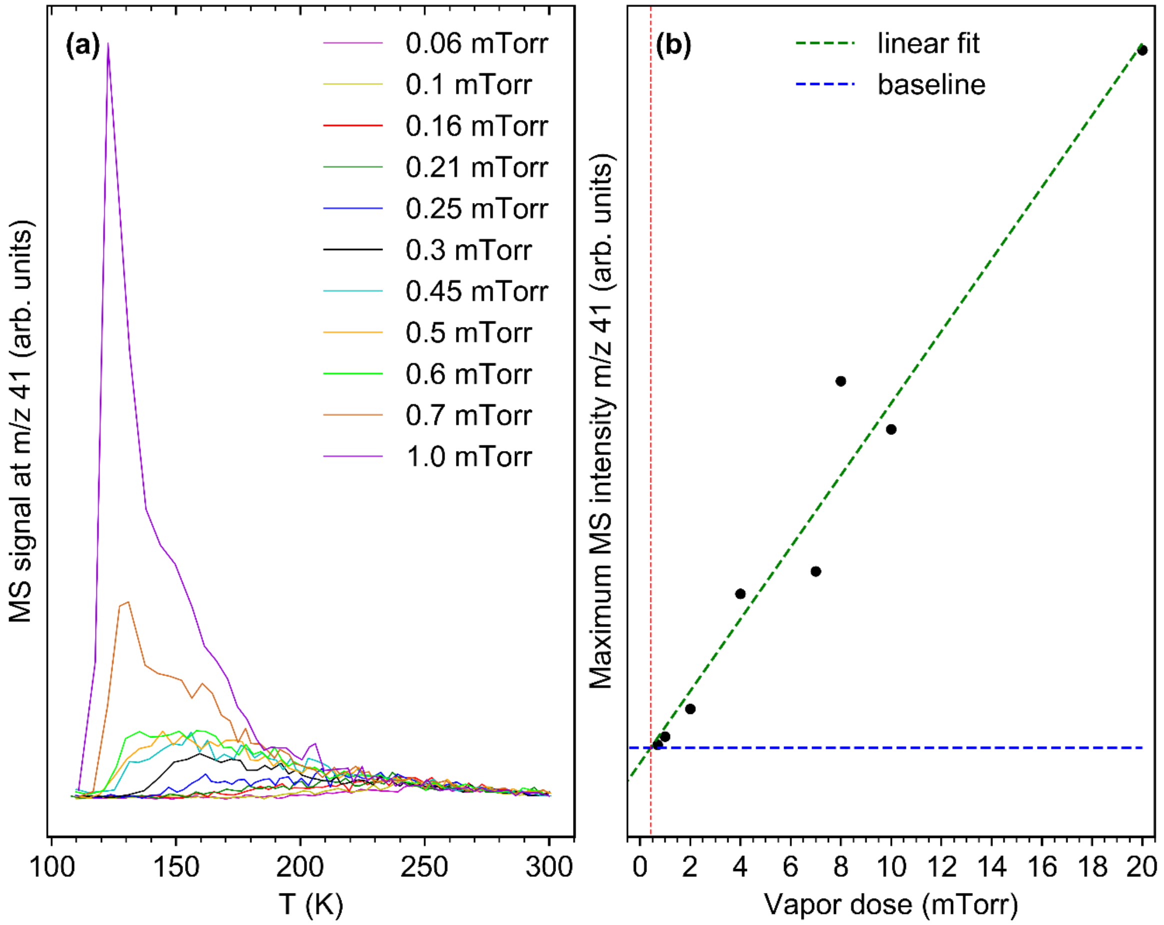

Figure S2. (a) Thermal desorption spectra of thin layers of 2M2B recorded after dosing various amounts of vapor to the Ta sheet held at 110 K. m/z 41 ($C_3H_5^+$) was chosen as a representative fragment of 2M2B. The multilayer desorption signal with maximum in the 120-130 K range started to emerge between vapor doses of 0.25 mTorr and 0.6 mTorr this giving evidence that the monolayer was saturated in this regime. (b) Height of the multilayer desorption signal of 2M2B for larger amounts of vapor dosed to the Ta sheet held at 110 K. The intersection of the linear fit to these data (green) with the baseline (blue) corresponds to a vapor dose of 0.43 mTorr, which falls in the range deduced in (a) and was consequently assigned to the pressure drop in the gas manifold that leads to monolayer coverage of 2M2B on the Ta substrate.

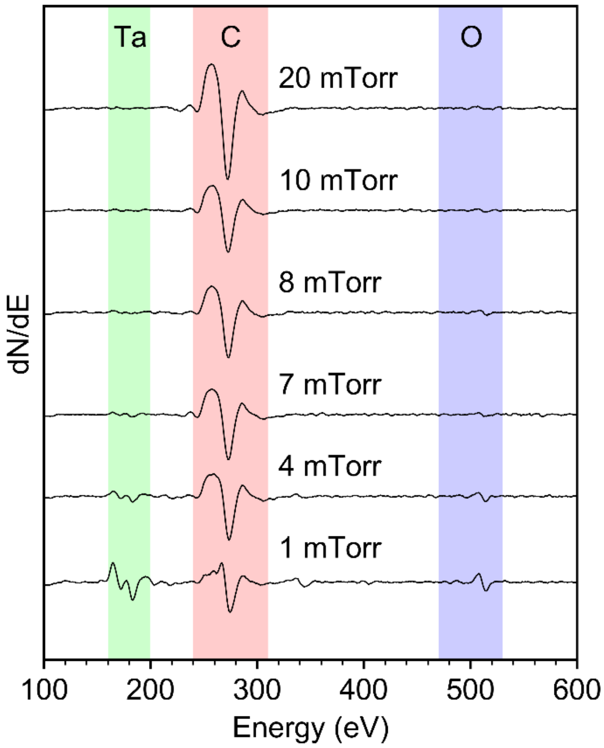

Figure S3. AES of deposits prepared from different amounts of adsorbed 2M2B by applying an electron exposure of 40 mC/cm^2 at E_0 = 31 eV (from bottom to top).

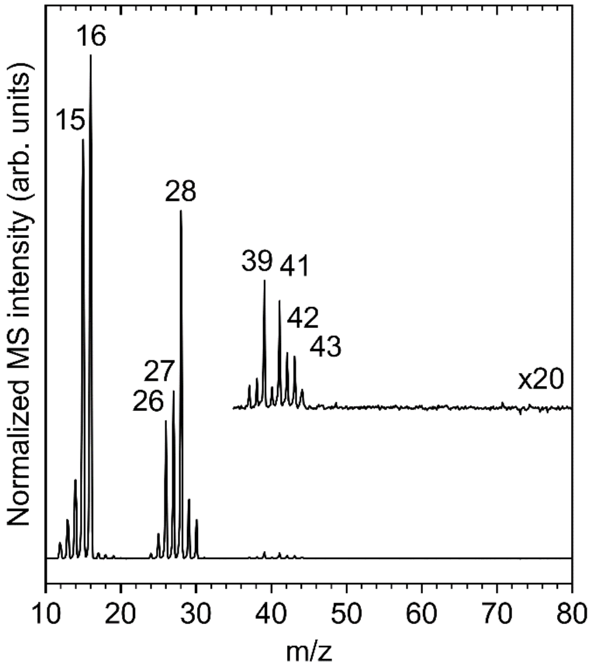

Figure S4. Mass spectrum recorded during electron exposure at E_0 = 31 eV of an adsorbed layer of *n*-pentane with thickness of 69 ML on a Ta substrate held at 110 K.

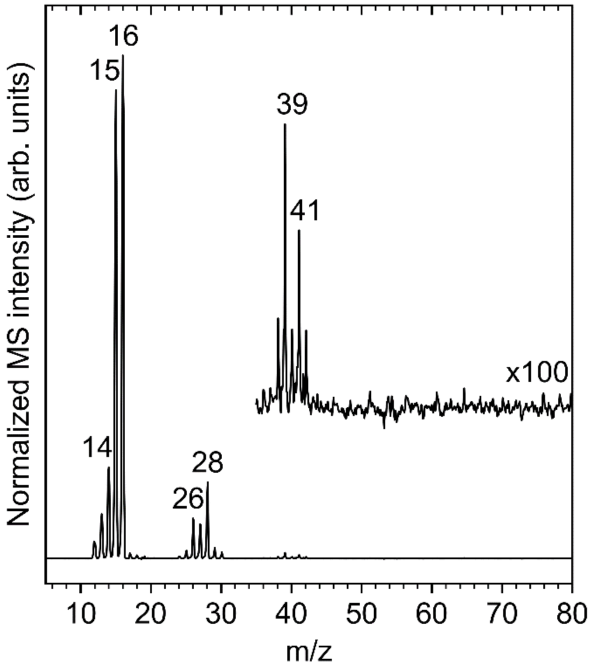

Figure S5. Mass spectrum recorded during electron exposure at E_0 = 31 eV of an adsorbed layer of 2M2B with thickness of 47 ML on a Ta substrate held at 110 K.

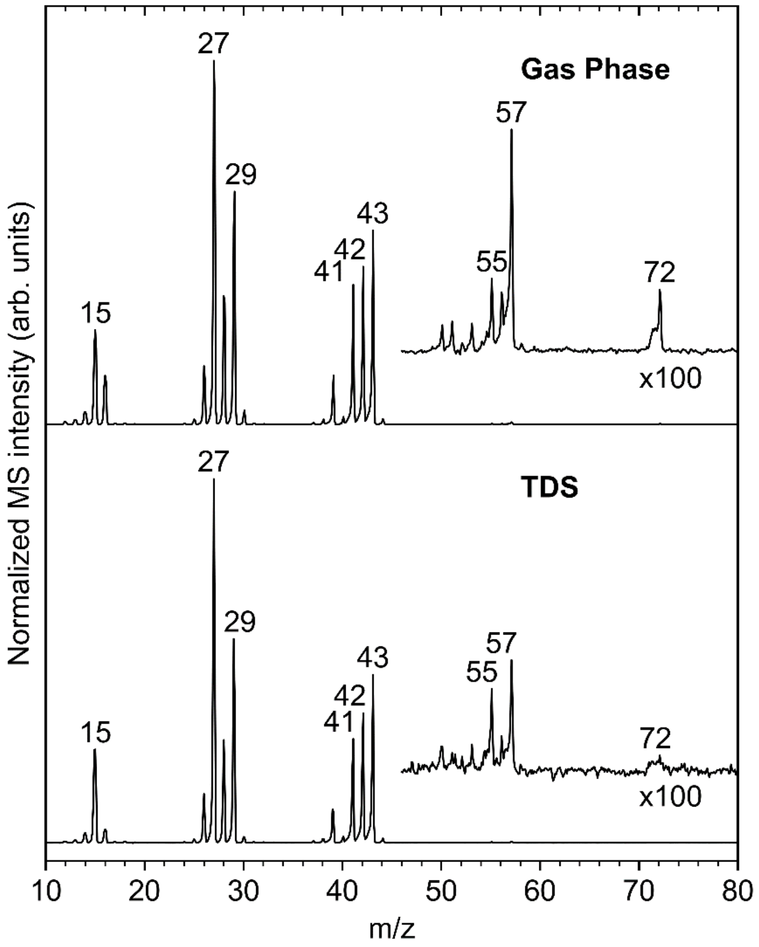

Figure S6. Mass spectrum recorded (top) upon dosing of *n*-pentane into the UHV setup and (bottom) in the temperature range 119-143 K during TDS of an adsorbed layer of *n*-pentane with thickness of 69 ML on a Ta substrate after an electron exposure of 40 mC/cm^2 at E_0 = 31 eV.

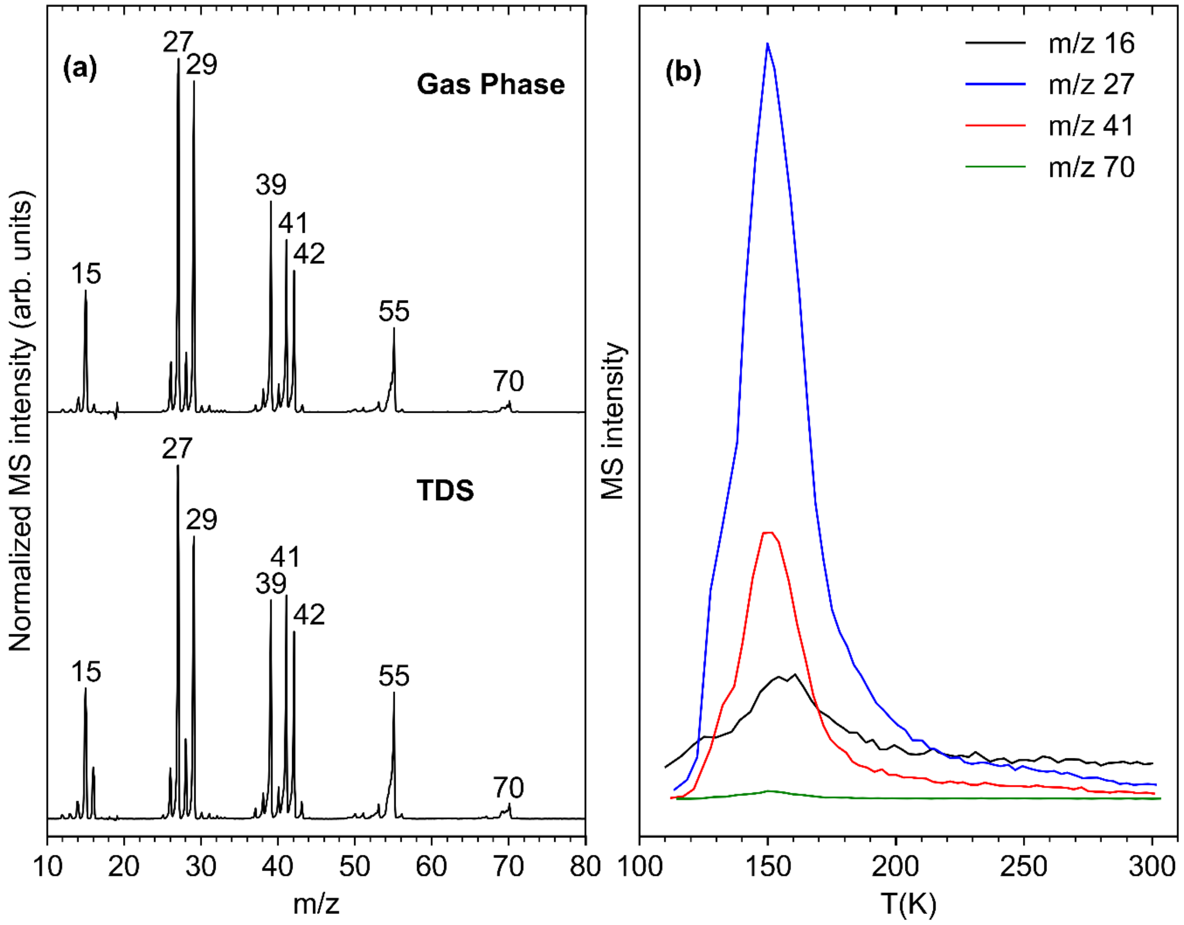

Figure S7. (a) Mass spectrum recorded (top) upon dosing of 2M2B into the UHV setup and (bottom) in the temperature range 121-144 K during TDS of an adsorbed layer of 2M2B with thickness of 47 ML on a Ta substrate after an electron exposure of 40 mC/cm^2 at E_0 = 31 eV. (b) TD spectrum of characteristic m/z traces after electron exposure.

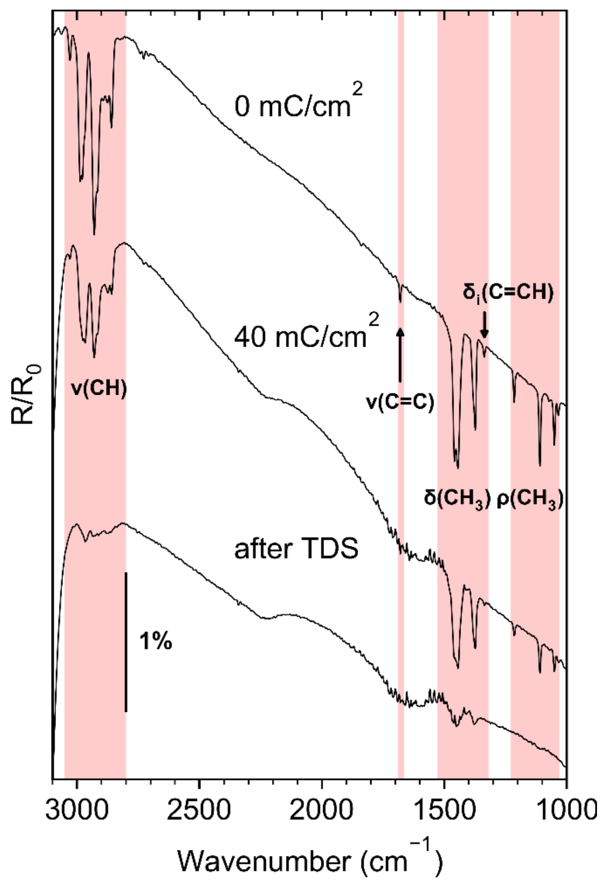

Figure S8. RAIR spectra of a pristine 47 ML 2M2B film on a Ta substrate held at 110 K directly after precursor dosing (0 mC/cm^2), after electron irradiation at E_0 = 31 eV (40 mC/cm^2), and after a subsequent annealing to 450 K. RAIRS acquired from the clean Ta sheet just before 2M2B dosing served as a background for all spectra shown here. The infrared spectrum of the precursor shows four major regions of signals (marked in red): Aliphatic and olefinic C–H stretches (ν(CH)) between 2800 and 3100 cm^{-1}, olefinic C–C stretches (ν(C=C)), bending vibrations of the terminal –CH_3 and C=CH groups (δ) between 1300 and 1500 cm^{-1} and CH_3 rocking vibrations (ρ) below 1200 cm^{-1}.

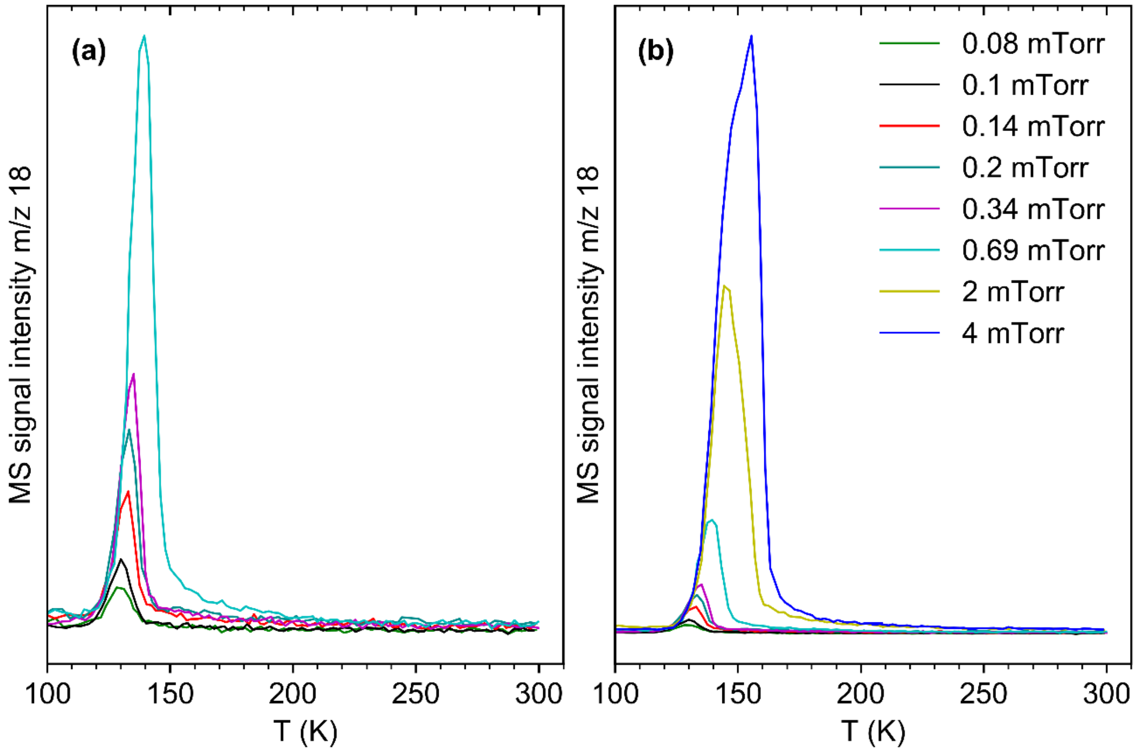

Figure S9. Thermal desorption spectra of H_2O (m/z 18) recorded after dosing various amounts of vapor onto a freshly prepared deposit from 69 ML *n*-pentane held at 110 K (a) excluding and (b) including high H_2O coverages.

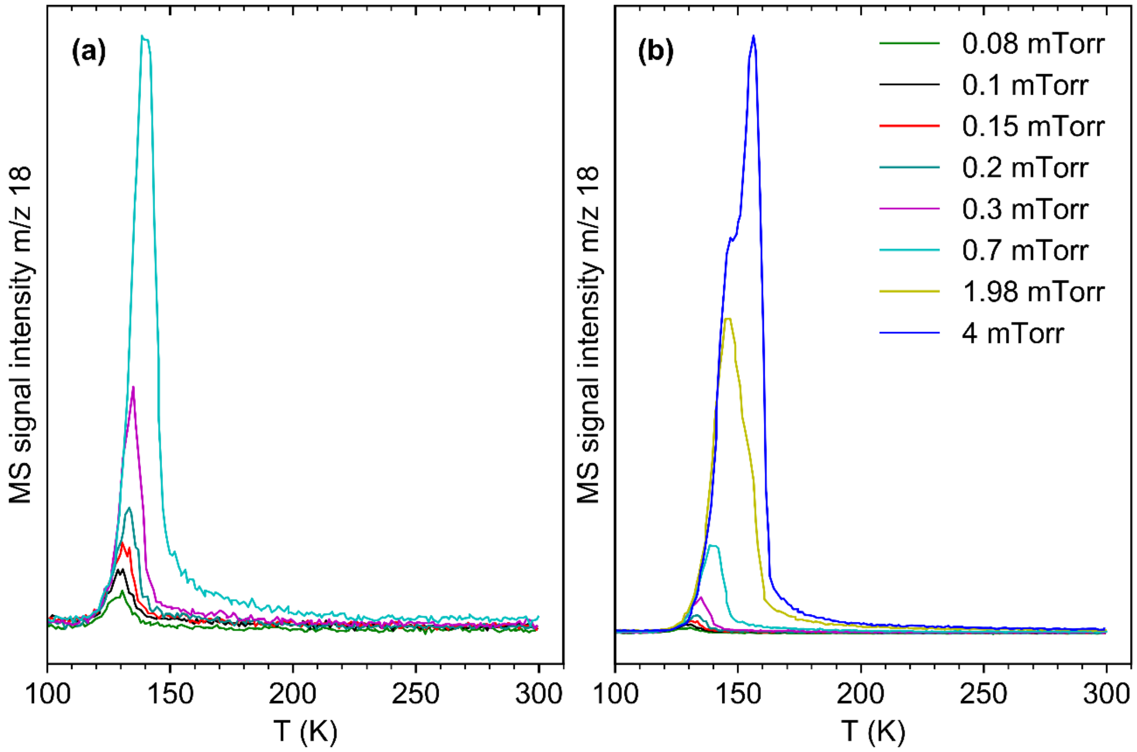

Figure S10. Thermal desorption spectra of H_2O (m/z 18) recorded after dosing various amounts of vapor onto a freshly prepared deposit from 47 ML 2M2B held at 110 K (a) excluding and (b) including high H_2O coverages.

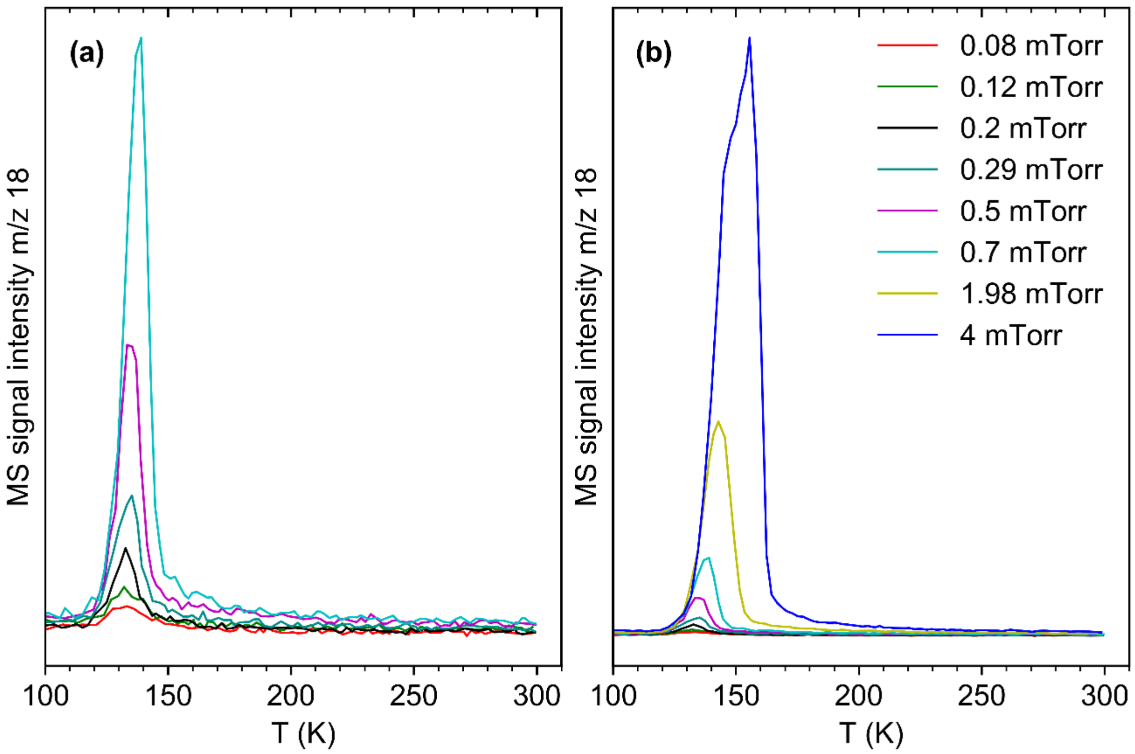

Figure S11. Thermal desorption spectra of H_2O (m/z 18) recorded after dosing various amounts of vapor onto a freshly prepared deposit from 8 ML $(EtCp)_2Ru$ held at 110 K (a) excluding and (b) including high H_2O coverages.

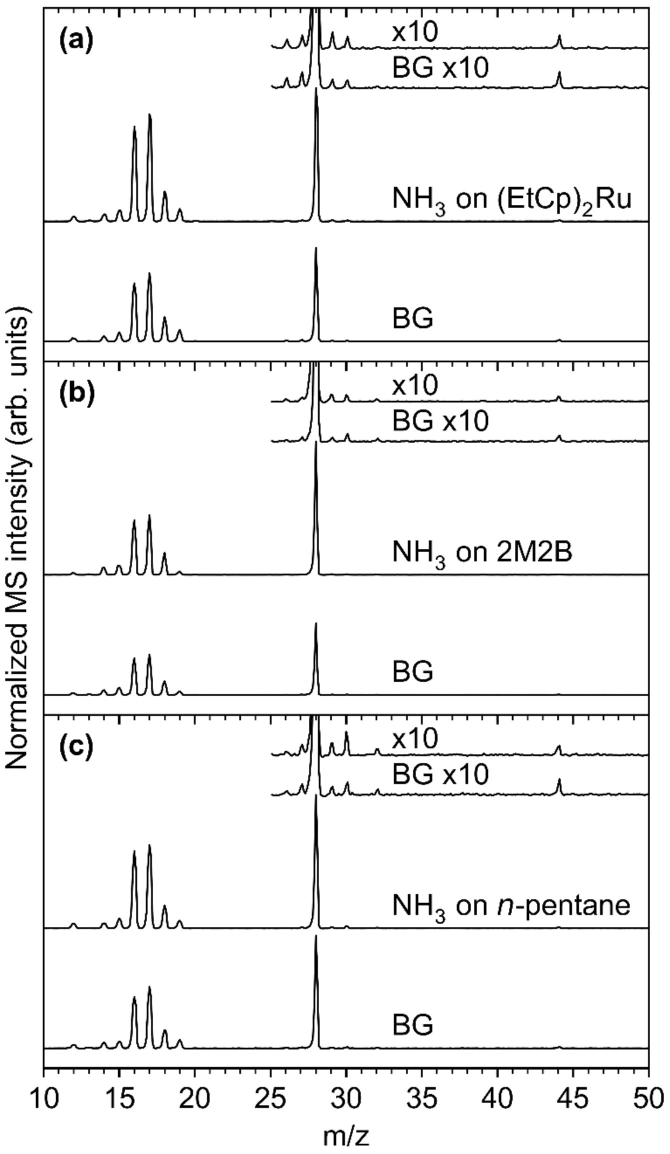

Figure S12. Mass spectra (m/z 10−50) of the volatile species produced upon ESD (E_0 = 31 eV) of an NH_3 film condensed on top of model deposits fabricated by electron irradiation (E_0 = 31 eV, 40 mC/cm^2) from (a) $(EtCp)_2Ru$, (b) 2M2B, and (c) *n*-pentane on a Ta substrate held at 110 K and subsequent annealing (450 K). The respective background mass spectrum (BG) recorded without electron irradiation is shown below each ESD mass spectrum. All ESD mass spectra of the deposits with adsorbed NH_3 are normalized to m/z 28. The corresponding mass spectra of the background are shown on the same scale as the ESD data.

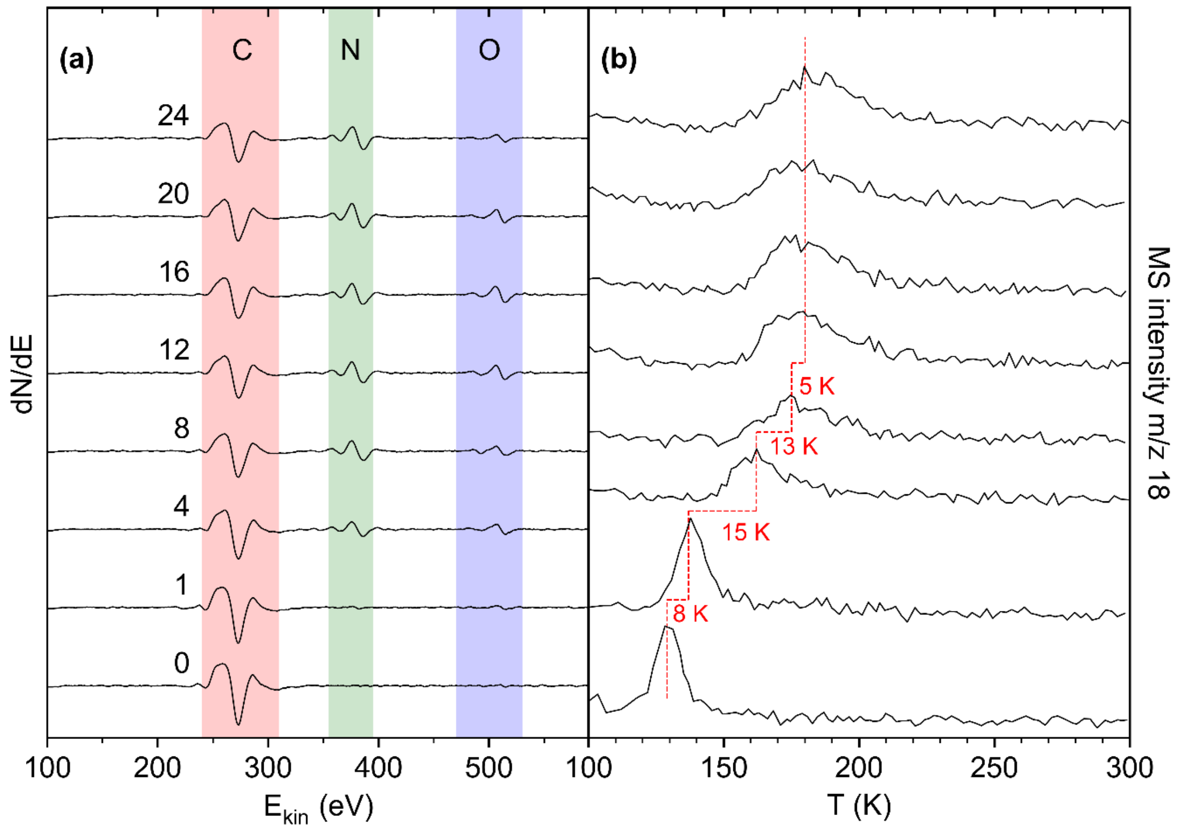

Figure S13. (a) AES of pristine deposits prepared from *n*-pentane (bottom spectrum in each panel) and of the same deposits after increasing number of treatment cycles by electron irradiation (40 mC/cm^2 at $E_0 = 31$ eV) in presence of NH_3 (4 mTorr). (b) Respective TDS acquired at m/z 18 ($H_2O^{\bullet+}$) after the same treatment cycles and subsequent dosing of H_2O (0.08 mTorr) onto the deposits prepared from *n*-pentane. The dashed red lines serve as a guide to the eye to facilitate observing shifts of the H_2O desorption signal. Temperature shifts between the presented cycles are denoted in red next to the respective TD spectra.

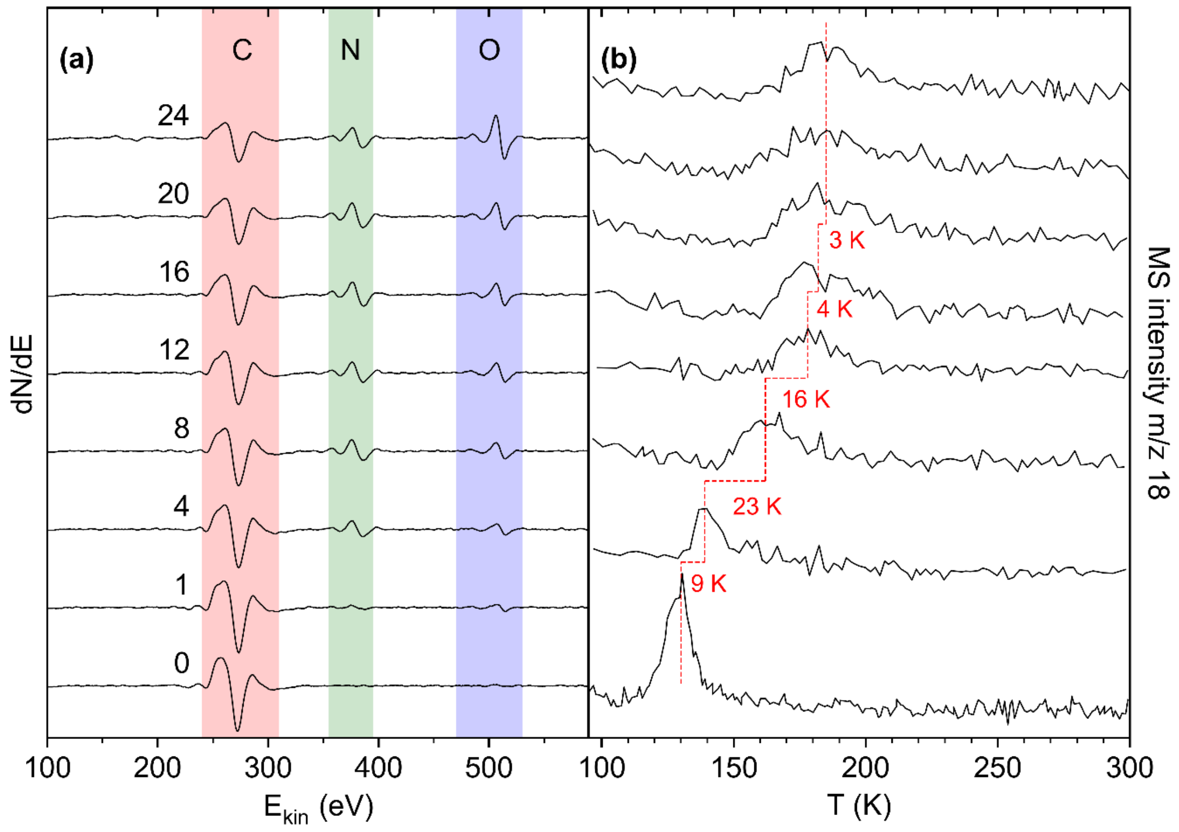

Figure S14. (a) AES of pristine deposits prepared from 2M2B (bottom spectrum in each panel) and of the same deposits after increasing number of treatment cycles by electron irradiation (40 mC/cm^2 at E_0 = 31 eV) in presence of NH_3 (4 mTorr). (b) Respective TDS acquired at m/z 18 ($H_2O^{\bullet+}$) after the same treatment cycles and subsequent dosing of H_2O (0.08 mTorr) onto the deposits prepared from 2M2B. The dashed red lines serve as a guide to the eye to facilitate observing shifts of the H_2O desorption signal. Temperature shifts between the presented cycles are denoted in red next to the respective TD spectra.

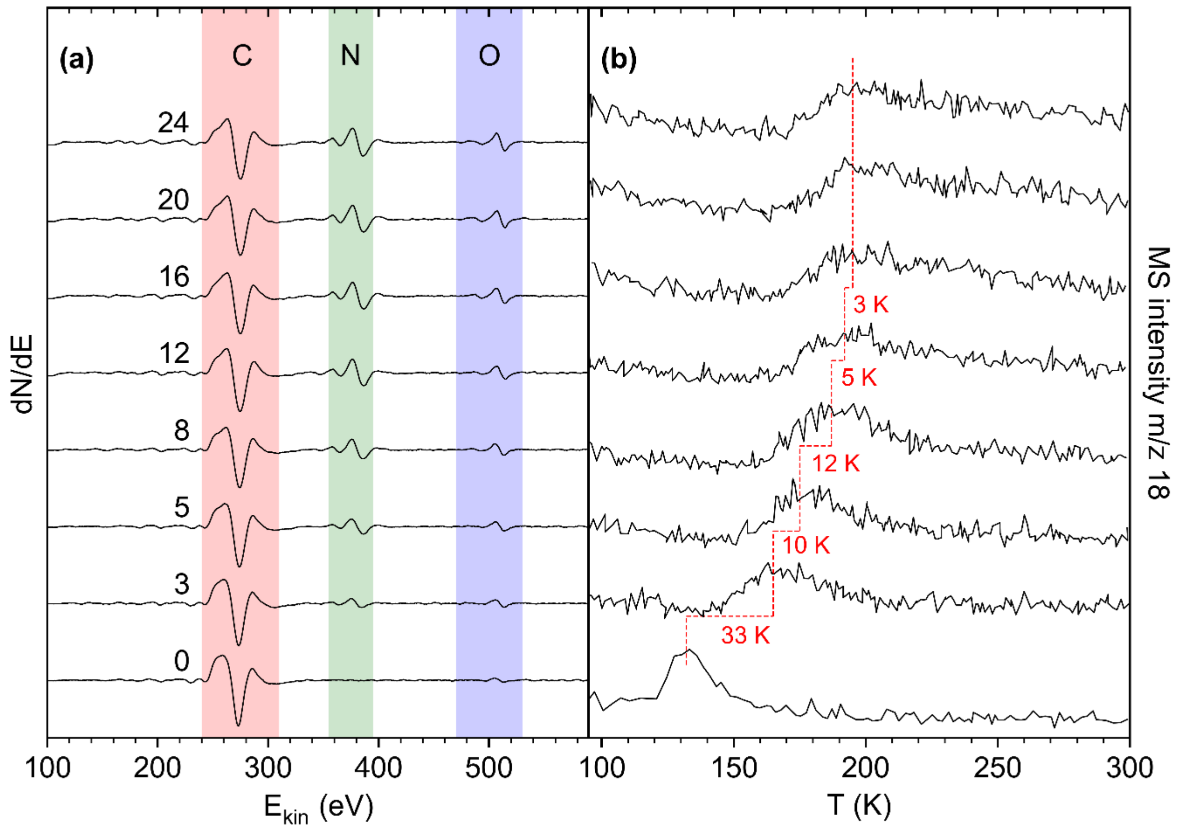

Figure S15. (a) AES of pristine deposits prepared from $(EtCp)_2Ru$ (bottom spectrum in each panel) and of the same deposits after increasing number of treatment cycles by electron irradiation (40 mC/cm^2 at E_0 = 31 eV) in presence of NH_3 (4 mTorr). (b) Respective TDS acquired at m/z 18 ($H_2O^{\bullet+}$) after the same treatment cycles and subsequent dosing of H_2O (0.08 mTorr) onto the deposits prepared from $(EtCp)_2Ru$. The dashed red lines serve as a guide to the eye to facilitate observing shifts of the H_2O desorption signal. Temperature shifts between the presented cycles are denoted in red next to the respective TD spectra.

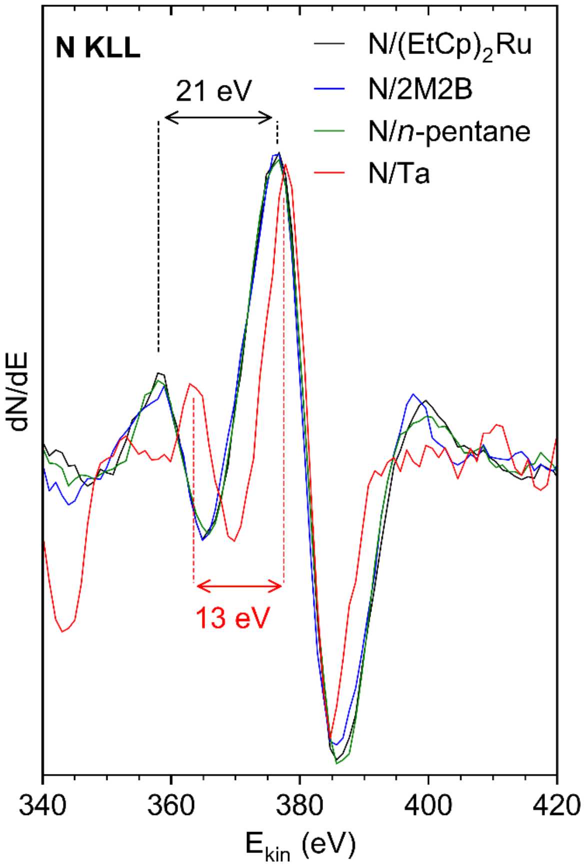

Figure S16. Enlarged derivative AES of the N KLL transition of the different deposits after a total of 24 NH_3 and electron treatment cycles (black: $(EtCp)_2Ru$, blue: 2M2B, green: *n*-pentane) and of the bare Ta substrate after exposure to exactly the same procedure (red curve).

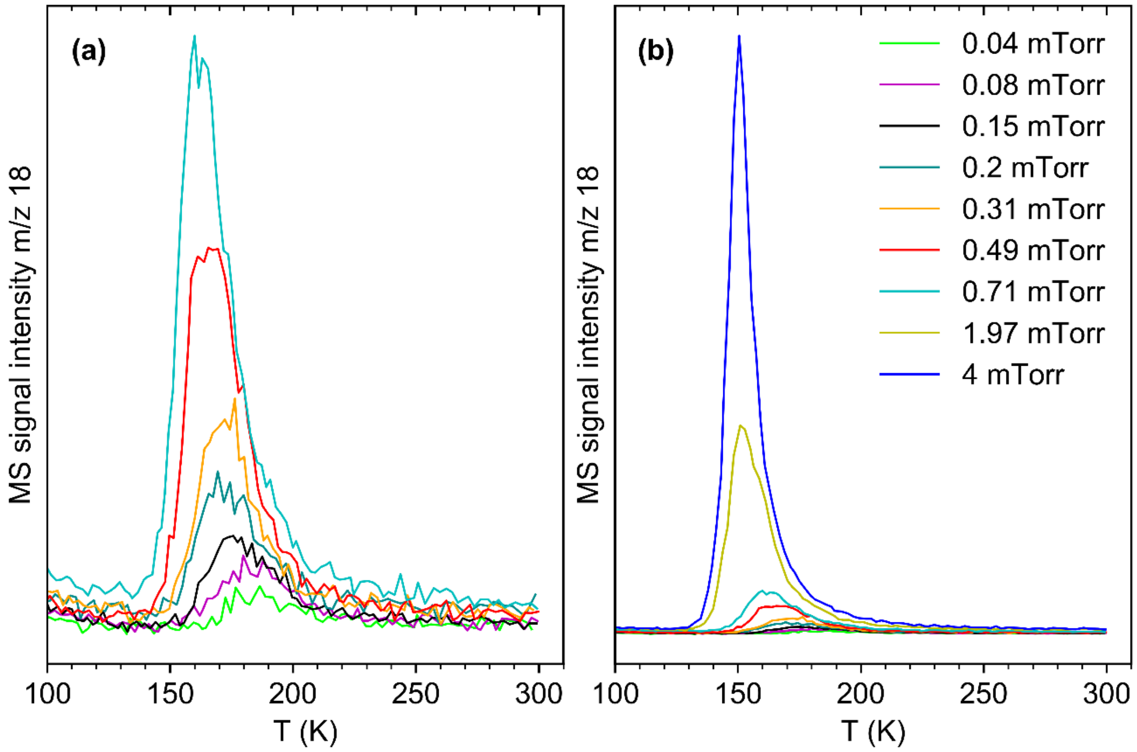

Figure S17. Thermal desorption spectra of H_2O (m/z 18) recorded after dosing various amounts of vapor onto a deposit prepared from 69 ML *n*-pentane held at 110 K after 24 NH_3 and electron treatment cycles (a) excluding and (b) including high H_2O coverages.

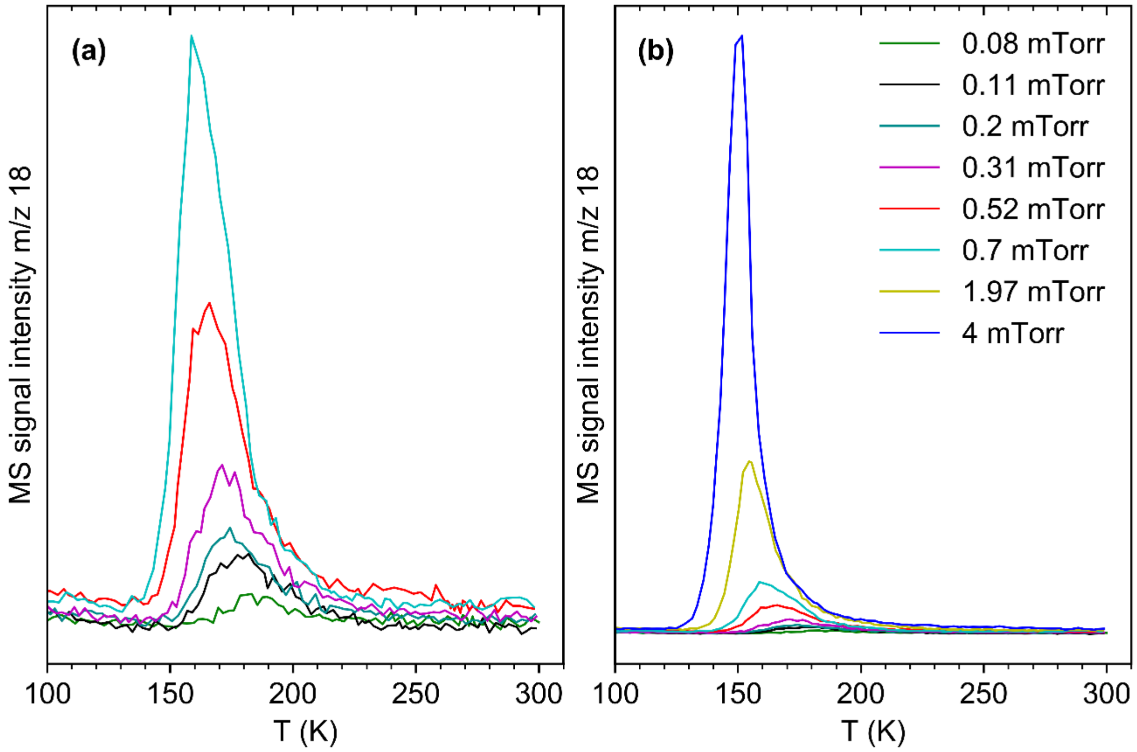

Figure S18. Thermal desorption spectra of H_2O (m/z 18) recorded after dosing various amounts of vapor onto a deposit prepared from 47 ML 2M2B held at 110 K after 24 NH_3 and electron treatment cycles (a) excluding and (b) including high H_2O coverages.

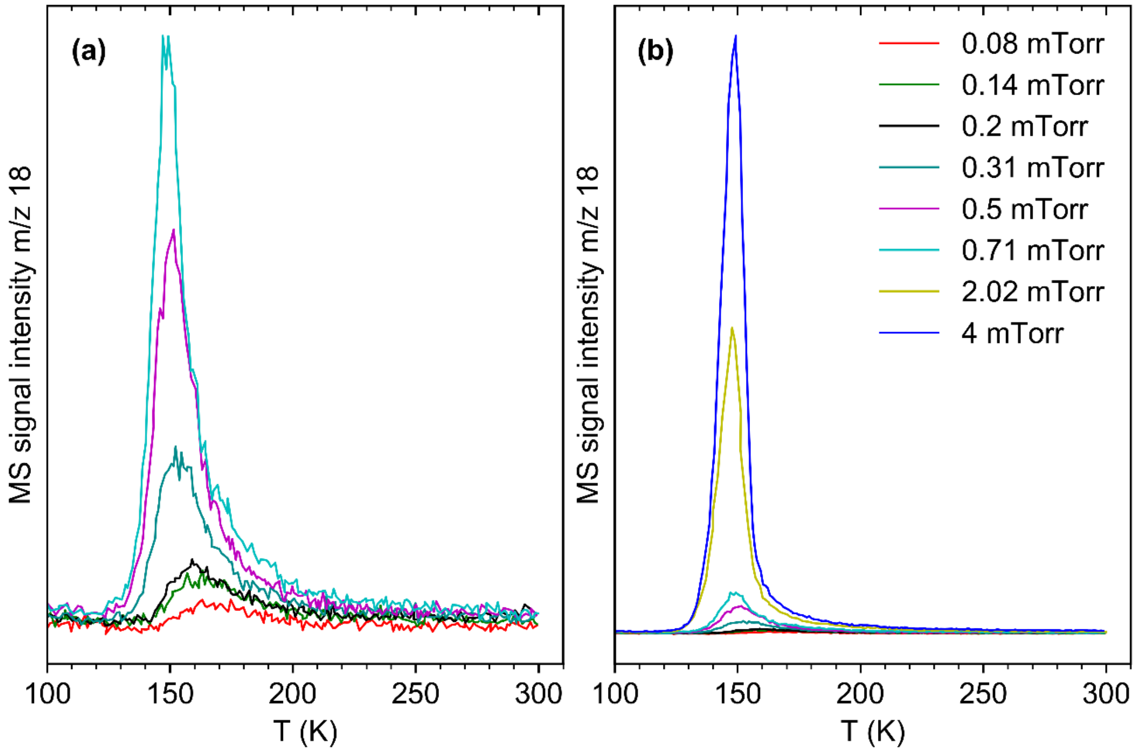

Figure S19. Thermal desorption spectra of H_2O (m/z 18) recorded after dosing various amounts of vapor onto a deposit prepared from 8 ML $(EtCp)_2Ru$ held at 110 K after 3 NH_3 and electron treatment cycles (a) excluding and (b) including high H_2O coverages.

Table S1. Evaluation of the C:N elemental ratio in the deposit prepared from n-pentane after increasing number of treatment cycles by electron irradiation (40 mC/cm^2 at E_0 = 31 eV) in presence of NH_3 (4 mTorr). The Peak-to-Peak (PtP) heights from the AES data were corrected by tabulated sensitivity factors at 5 keV (S_C = 0.4763, S_N = 0.9157).

Cycle	PtP height C	PtP height C / S_C	PtP height N	PtP height N / S_N	C:N ratio
1	21443	49229	1058	1155	1:0.02
4	18701	45020	5634	6153	1:0.14
8	16651	39263	7519	8211	1:0.21
12	16115	34959	8004	8741	1:0.25
16	15432	32400	7869	8593	1:0.27
20	15884	33349	9313	10170	1:0.30
24	14950	31388	8634	9428	1:0.30

Table S2. Evaluation of the C:N elemental ratio in the deposit prepared from 2M2B after increasing number of treatment cycles by electron irradiation (40 mC/cm^2 at E_0 = 31 eV) in presence of NH_3 (4 mTorr). The Peak-to-Peak (PtP) heights from the AES data were corrected by tabulated sensitivity factors at 5 keV (S_C = 0.4763, S_N = 0.9157).

Cycle	PtP height C	PtP height C / S_C	PtP height N	PtP height N / S_N	C:N ratio
1	27603	57953	1712	1870	1:0.03
4	24030	50451	6005	6558	1:0.13
8	22094	46387	7814	8533	1:0.18
12	20482	43002	7807	8526	1:0.20
16	19261	40439	9229	10079	1:0.25
20	17623	37000	9525	10402	1:0.28
24	15031	31558	7675	8381	1:0.27

Table S3. Evaluation of the C:N elemental ratio in the deposit prepared from $(EtCp)_2Ru$ after increasing number of treatment cycles by electron irradiation (40 mC/cm^2 at E_0 = 31 eV) in presence of NH_3 (4 mTorr). The Peak-to-Peak (PtP) heights from the AES data were corrected by tabulated sensitivity factors at 5 keV (S_C = 0.4763, S_N = 0.9157, S_{Ru} = 1.8454). [1] The contribution of C to the overlapping C KLL and Ru MNN Auger signals around 275 eV was estimated based on these factors as described previously [2] by assuming that the typical composition of a deposit prepared by electron irradiation of $(EtCp)_2Ru$ has a typical composition between RuC_9 and RuC_{14}, the latter corresponding to the elemental ratio of the pristine precursor. This yields lower and upper limits to the PtP height of C KLL alone of 70% and 78% of the overlapping C KLL and Ru MNN signals listed as third column of the table after correction by the sensitivity factor S_C.

Cycle	PtP height C:Ru	PtP height C / S_C<br>Lower limit<br>Upper limit	PtP height N	PtP height N / S_N	C:N ratio<br>Upper<br>Lower
1	34479	50672<br>56464	4650	5078	1:0.10<br>1:0.09
4	33023	48533<br>54079	7738	8450	1:0.17<br>1:0.16
8	31341	46061<br>51325	10164	11089	1:0.24<br>1:0.22
12	32784	48181<br>53688	13675	14934	1:0.31<br>1:0.28
16	31681	46560<br>51882	13911	15192	1:0.33<br>1:0.29
20	30786	45245<br>50416	13809	15080	1:0.33<br>1:0.30
24	31374	46109<br>51379	14469	15801	1:0.34<br>1:0.31

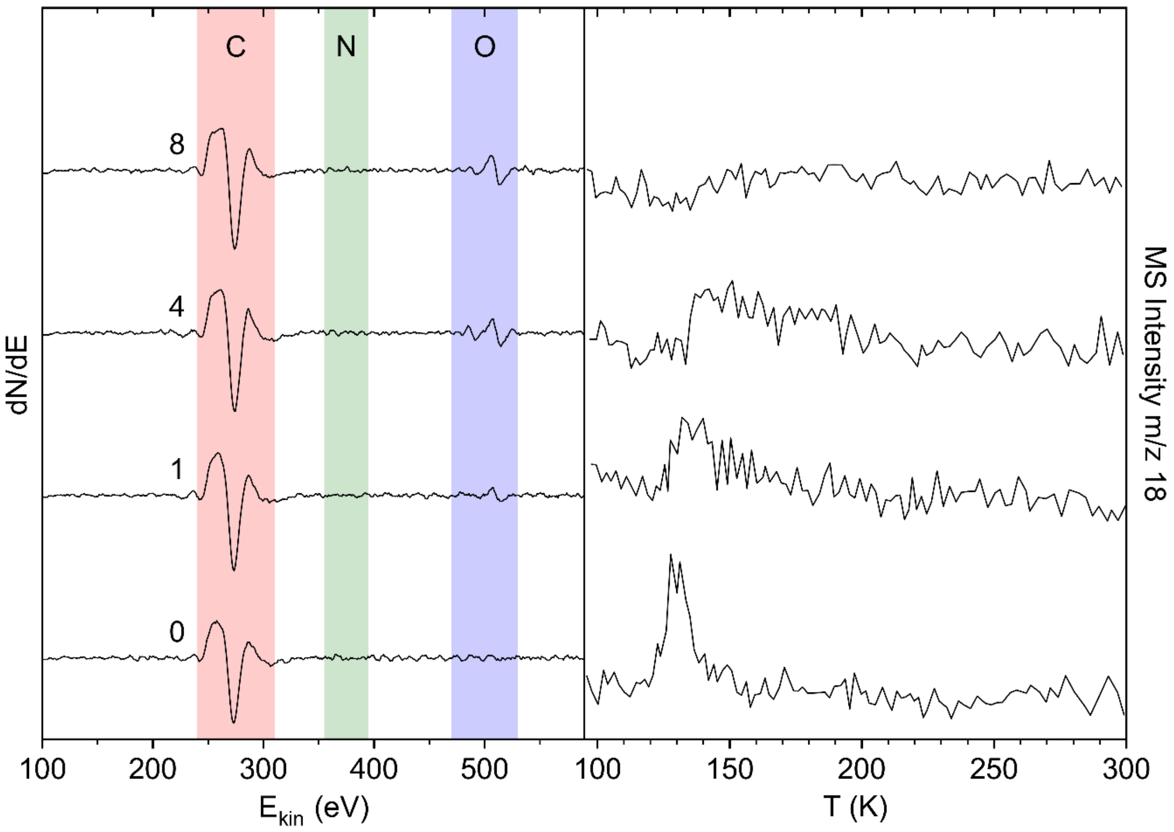

Figure S20. AES of pristine deposits prepared from 2M2B (bottom spectrum in each panel) and of the same deposits after increasing number of treatment cycles by electron irradiation (40 mC/cm^2 at E_0 = 31 eV). Respective TDS acquired at m/z 18 ($H_2O^{\bullet +}$) after the same treatment cycles and subsequent dosing of H_2O (0.08 mTorr) onto the deposits prepared from 2M2B.

References

1. Briggs, D.; Grant, J.T. (Eds.) *Surface Analysis by Auger and X-ray Photoelectron Spectroscopy*; SurfaceSpectra: Manchester, England, 2003; ISBN 1901019047.
2. Rohdenburg, M.; Winkler, R.; Kuhness, D.; Plank, H.; Swiderek, P. Water-Assisted Process for Purification of Ruthenium Nanomaterial Fabricated by Electron Beam Induced Deposition. *ACS Appl. Nano Mater.* **2020**, *3*, 8352–8364. https://doi.org/10.1021/acsanm.0c01759.
